# Utility of fatty acid profile and in vitro immune cell activation for chemical and biological standardization of *Arthrospira*/*Limnospira*

**DOI:** 10.1038/s41598-022-19590-x

**Published:** 2022-09-19

**Authors:** Jungmoo Huh, Jin Zhang, Radka Hauerová, Joseph Lee, Saqlain Haider, Mei Wang, Tomáš Hauer, Ikhlas A. Khan, Amar G. Chittiboyina, Nirmal D. Pugh

**Affiliations:** 1 National Center for Natural Products Research, Research Institute of Pharmaceutical Sciences, School of Pharmacy, The University of Mississippi, University, MS 38677 USA; 2 Faculty of Science, University of South Bohemia, Branišovská 1760, 370 05 České Budějovice, Czech Republic; 3 Natural Products Utilization Research Unit, Agricultural Research Service, United States Department of Agriculture, University, MS 38677 USA; 4 Department of BioMolecular Sciences, School of Pharmacy, The University of Mississippi, University, MS 38677-1848 USA

**Keywords:** RNA sequencing, Toll-like receptors, Natural products, Fatty acids, Chemical tools

## Abstract

Commercially cultivated *Limnospira* (species formerly classified to genus *Arthrospira*) is a popular food/supplement consumed by millions of people worldwide for health benefits. The objective of the current research was to advance the standardization technology for *Limnospira*. Quantitative methods were established to detect fatty acids as potential chemical markers and immune-enhancing activity. Analysis of 20 different batches of biomass obtained from one commercial grower demonstrated that there was a statistically significant relationship between the sum of two fatty acids (linoleic and γ-linolenic) and Toll-like receptor (TLR)2/TLR1-dependent activation (*R*^2^ = 0.48, *p* = 0.0007). Investigation of 12 biomass samples sourced from growers in 10 different countries demonstrated that fatty acid content was again significantly correlated with biological activity (*R*^2^ = 0.72, *p* = 0.0005) and the content of fatty acids varied by twofold and activity by 12.5-fold. This large variation between different samples confirms the need to use the present standardization methods to ensure consistent and properly characterized biomass for consumers and for future scientific research.

## Introduction

Authentication and standardization have emerged as two critical tools for the rigorous scientific evaluation of botanical dietary supplements. Botanical authentication establishes the complete taxonomic identification and provides a foundation for future studies on the same test material. Lack of properly authenticated study material has resulted in published work that is misleading and potentially not reproducible^1^. Additionally, misidentification and adulteration with other species can impact the efficacy and safety of a botanical for consumers^2^. The objective of botanical standardization is to ensure that product material exhibits consistent and known levels of the biologically active compounds. Evaluation of material that is not standardized can be a major issue since botanicals often exhibit substantial variation in the level of their chemical constituents^2^, and this has contributed to a lack of consistent product efficacy in clinical trials^3^.

In the last 30 years several authors confirmed classification of *Arthrospira* and *Spirulina* into two independent genera, which was more or less widely accepted^4,5^. However, *Arthrospira* biomass is still widely referred to as “spirulina” by the general public, commercial marketing, and occasionally in the scientific literature. The use of incorrect taxonomic nomenclature can create confusion for data interpretation. In addition, cases of incorrect species designations within culture collections have also contributed to confusion regarding the correct identification of *Arthrospira* biomass material^6^. Recent taxonomic advances have resulted from investigating the *Arthrospira* type species based on a polyphasic approach using morphology, ecology, cell wall ultrastructure, and 16S rRNA sequence analysis. Based on this detailed characterization, *Limnospira* has been proposed as a new genus containing species formally part of *Arthrospira* and including the commercially grown taxa used for food and botanical dietary supplements^6^. Thus, in this paper, we refer to this organism by its taxonomically correct genus name—*Limnospira*.

Certain cyanobacterial representatives are used for human consumption^7^. *Arthrospira/Limnospira* is among the cyanobacterial genera that have been used as food for hundreds of years. Its species have been harvested in alkaline lakes, for example in Africa around Lake Chad and by the Aztecs in Central Mexico under the names dihé or tecuitlatl, respectively^8^. Since 1973, when a pilot plant in Mexico started annual production of 150 tons of dry biomass^9^, it has been cultivated on an industrial scale under control conditions in numerous places across the globe. The product is usually marketed under the name Spirulina. Although early interest in commercializing this food was focused mainly on its nutrient and protein content, it has emerged as a popular botanical dietary supplement exhibiting various human health benefits. A growing body of evidence indicates that this botanical’s consumption is a particularly useful natural product with immune-enhancing benefits that provides resilience against respiratory viral infection^10–12^.

Despite the popularity of *Limnospira* as a botanical dietary supplement, little work has been achieved on standardization methods. In the US Pharmacopeia method^13^, fatty acid profile, chlorophyll A and carotenoids are listed as chemical entities useful for identification purposes. For composition, the USP method includes the content of β-carotene, total carotenoids, c-phycocyanin, and protein. Since many of these constitutents are also present within other natural products, it remains to be determined if any of these can be used as meaningful authentication markers and whether any are correlated with *Limnospira*-dependent activation of immune cells, a major biological activity attributed to this botanical^14^.

Previous studies identified Braun-type lipoproteins as the predominant macrophage-activating principal within *Limnospira*^14^. Based on this research, a botanical extract was commercially developed that preferentially enriches the level of these active macromolecules (trade name Immulina). Currently, Immulina is standardized using only an in vitro bioassay that quantitates general monocyte-activation (without specificity for receptor mechanism of action). Chemical standardization of macromolecules, such as lipoproteins, can be challenging because the biological activity of these substances is often poorly correlated to their chemical composition. A similar problem existed in pharmaceutical biologics, a problem eventually solved by bioassay-based standardization to afford the consistent quality of products. Therefore, proper characterization of biologics requires information from both chemical and bioassay methods^15^.

The objective of the present research was to advance the standardization technology for *Limnospira* botanical dietary supplements and food products by employing a combination of chemical- and bioassay-based approaches. Selection and establishment of quantitative methods employed the use of biomass that was taxonomically identified using the most current classification system. Since the lipid moiety is the integral structural component on Braun-type lipoproteins, we hypothesized that the fatty acid profile could be a potential chemical standardization marker predictive of immune-enhancing activity. To test this hypothesis, we evaluated 20 different lots of biomass obtained from one commercial grower as well as material sourced from commercial growers in 10 different countries.

## Results and discussion

### Taxonomic identification using sequence analysis and morphological examination

**Figure 1:**
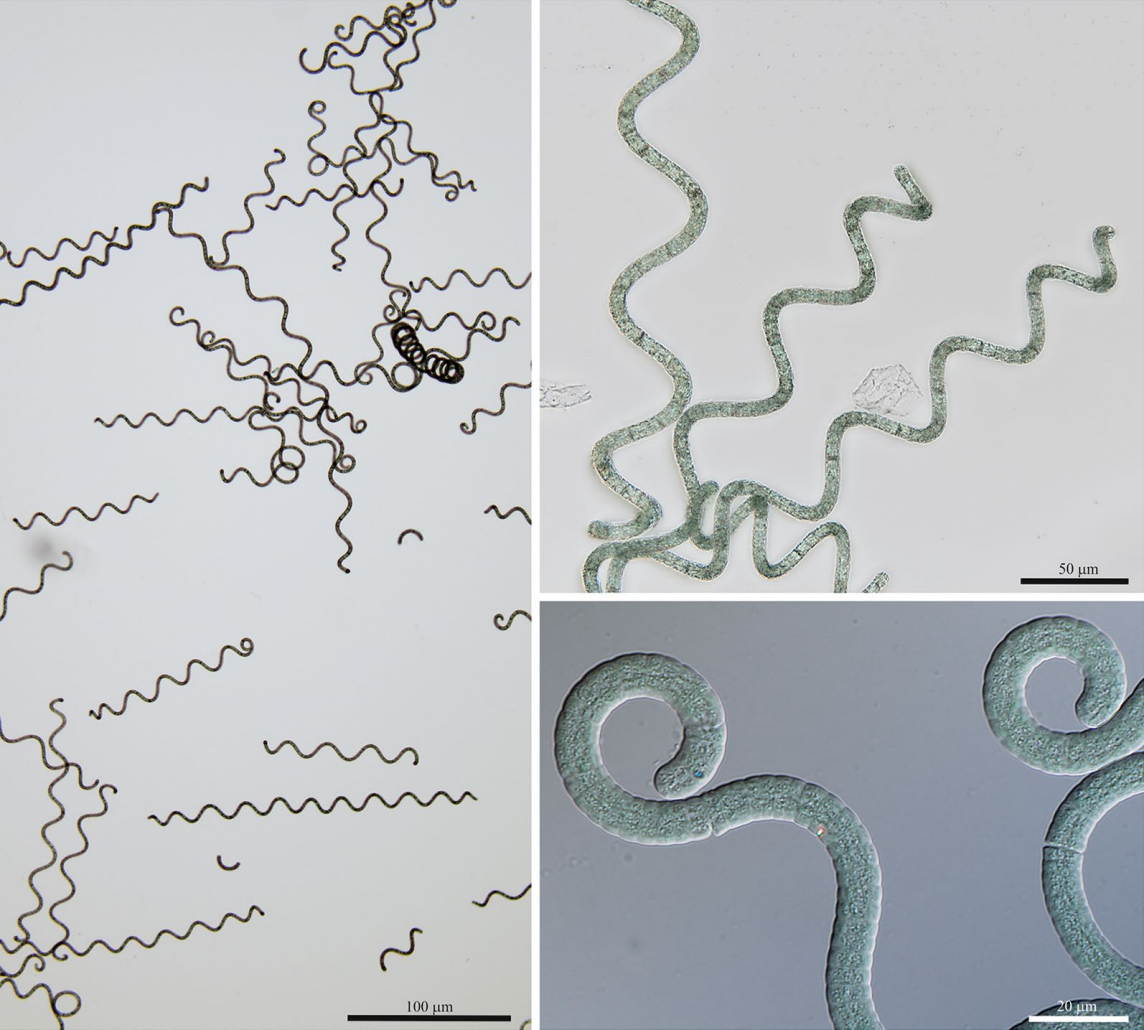
Morphology of material obtained from Dongtai Cibainian Biological Engineering Co. used as reference in this study.

According to its morphological features (Fig. 1) the reference study material (obtained from Dongtai Cibainian Biological Engineering) was determined using^16^ as *L. fusiformis*, but with opposite spirality than the one cited in^16^, i.e. sinistral instead of dextral*.* In terms of this character the species would be *L. maxima*, however the comparison of sequences of 16S rRNA gene + 16S-23S ITS region has shown accordance with genome sequence of reference strain of *L. fusiformis*, i.e. SAG 85.79*.* In any case this is a difference to the designation used by the manufacturer—*A. platensis*. Regardless of generic assignment which has undergone formal nomenclature change, the species are different based on their morphology. Unfortunately, it is not possible to apply molecular comparison because of lack of reference sequence of *A. platensis* in public sequence repositories. The sequences available bearing this name cannot be considered as reference, since the material was not isolated from a site similar to the *locus classicus* of *A. platensis*^17^ in terms of ecology and geography. In any case, usefulness of trichome spirality as one of the features for species determination needs to be reviewed based strictly on reference material. The same applies on taxonomic assignment of sequence data available in public repositories. However, for such studies availability of reference strain of at least *A. platensis* is a necessity.

The analysis of powdered biomass sold as food supplement (Table 1) revealed that the microscopy is rather difficult tool for species delimitation, since post-harvest processing of the material leads to biomass disintegration in some extent. These steps cause loss of some important morphological features like style of trichome coiling, or their well-developed terminal parts. The microscopic appearance of powdered biomass may be influenced also by length of its storage. Additionally, the DNA of the powdered biomass was extracted and subsequently the 16S rRNA genes of the sold food supplements were sequenced. The obtained sequences were compared to the sequences extracted from the genome of the reference strain of the type species of the *Limnospira* genus—*L. fusiformis* SAG 85.79, as well as to the outgroup taxon *Limnoraphis hieronymusii* CN4-3 AB045906 that was shown in previous phylogenetic studies to be closely related to the genus *Limnospira*^6^. Comparison of 16S rRNA gene identities among these sequences (Table 2) revealed that only the outgroup taxon considerably differed from the rest. On the other hand, all the taxa sequenced in this study shared more than 99% sequence identity with the reference 16S rRNA gene of the type species’ reference strain *L. fusiformis* SAG 85.79 and more than 98.8% identity among each other. For specimens of *Limnospira* 1, 3, 4, 5, 7, 8, 9, and 11 (numbers according to Table 1) sequences of the 16S rRNA gene were obtained from three clones. Our data suggest that the variability among different rRNA operons within the organisms is more noticeable than variability among the orgasnisms themselves; thus, the obtained data do not indicate that there is more than one species present in the food supplements investigated here. However, the high operon variability within the organisms makes the taxonomic situation of the genus *Limnospira* even more confusing. The taxonomic status and position of the species currently recognized in the genus *Limnospira* were not completely clarified yet and will need more thorough study using more molecular markers that is out of scope of the current paper.

**Table 1:** Variation in total fatty acid content and immune-enhancing activity (TLR2/TLR1 activation) in biomass samples obtained from various commercial growers throughout the world.

	Company	Country	Lot number	Biomass	Total fatty acids (mg/g)	TLR2/TLR1 (EC_50_ value)	16S rRNA, 16S-23S ITS
1	Cyanotech Corporation	USA	1400036543	*Arthrospira platensis*	44.91	5.99	OM419146–8
2	Earthrise Nutritionals	USA	41739	*Arthrospira platensis*	59.47	3.62	OM419149
3	Solarium Biotechnology S.A	Chile	not available	*Arthrospira maxima*	46.15	7.01	OM419150–2
4	TAAU Australia Pty Ltd	Australia	not available	*Arthrospira maxima*	35.83	45.29	OM419153-–5
5	FEBICO	Taiwan	20,200,309,001 02	*Spirulina platensis*	51.30	5.43	OM419156–8
6A	EID—Parry	India	SPEPF2001	*Arthrospira platensis*	50.77	6.65	OM419159
6B	EID—Parry (sold by Triquetra Health)	India	1952	*Arthrospira platensis*	48.15	18.72	OM419160
7	Dongtai Cibainian Biological Engineering	China	20190501	*Arthrospira platensis*	51.34	5.23	OM419161–3
8	Spirulina Nigrita	Greece	148	*Arthrospira platensis*	31.35	39.16	OM419164–6
9	Flora	Mongolia	180968	*Arthrospira platensis*	54.10	6.90	OM419167–9
10	Akal Food	France	2048/SOL	*Arthrospira platensis*	43.74	9.03	OM419170
11	Akal Food	Burkina Faso	2029	*Arthrospira platensis*	49.88	4.03	OM419171–3

Biomass identification is based on the company literature, 16S rRNA and 16S-23S ITS region sequences were obtained in this study. EC_50_ values represent the concentration (µg/mL) of biomass material required to induce activation for the TLR2/TLR1 signaling pathway to levels 50% of those achieved by Pam_3_CSK_4_ (100 ng/mL).

**Table 2:** Comparison of percentage of the 16S rRNA gene sequence identity among obtained *Limnospira* sequences, the reference strain of the type species *L. fusiformis* SAG 85.79 (CP051185), and an outgroup taxon *Limnoraphis hieronymusii* (AB045906).

	*Limnoraphis*	*L. fusiformis*	1	2	3	4	5	6a	6b	7	8	9	10
*L. fusiformis* SAG 85.79	94.6												
*Limnospira* 1	94.7–94.8	99.5–99.7											
*Limnospira* 2	94.9	99.9	99.4–99.5										
*Limnospira* 3	94.7–94.9	99.7–99.9	99.3–99.6	99.6–99.8									
*Limnospira* 4	94.6–94.8	99.7–99.9	99.2–99.6	99.6–99.8	99.5–99.9								
*Limnospira* 5	94.7–94.9	99.7–99.9	99.3–99.7	99.5–99.8	99.4–99.9	99.4–99.9							
*Limnospira* 6a	95.0	100	99.5–99.6	99.98	99.7–99.9	99.7–99.9	99.6–99.9						
*Limnospira* 6b	94.7	99.8	99.3–99.5	99.7	99.5–99.7	99.5–99.7	99.5–99.7	99.8					
*Limnospira* 7	94.7–94.8	99.8–99.9	99.3–99.6	99.7–99.8	99.5–99.9	99.5–99.9	99.5–99.9	99.8–99.9	99.6–99.7				
Limnospira 8	94.5–94.9	99.3–99.7	99.2–99.8	99.2–99.6	99.0–99.7	99.0–99.7	99.1–99.8	99.3–99.7	99.2–99.5	99.1–99.7			
*Limnospira* 9	94.5–94.7	99.5–99.9	99.0–99.5	99.5–99.7	99.2–99.8	99.2–99.8	99.2–99.8	99.6–99.9	99.3–99.7	99.3–99.8	98.8–99.6		
*Limnospira* 10	94.9	99.9	99.4–99.6	99.9	99.6–99.9	99.6–99.9	99.6–99.9	100	99.7	99.7–99.9	99.2–99.6	99.5–99.8	
*Limnospira* 11	94.5–94.8	99.6–99.9	99.1–99.6	99.8–99.8	99.3–99.9	99.3–99.9	99.2–99.9	99.6–99.9	99.4–99.7	99.4–99.9	98.9–99.7	99.1–99.8	99.5–99.9

Numbers at *Limnospira* names correspond to numbers at Table 1. For *Limnospira* 1, 3, 4, 5, 7, 8, 9, and 11 sequences of 3 clones of the 16S rRNA gene were obtained.

### Quantitative fatty acid profiling as a chemical standardization method for *Limnospira* biomass and immulina extract

Hyphenated analytical methods are instrumental and routinely implemented in the quality assurance of botanical extracts in various matrices. For example, detection of volatile organics in an extract with gas chromatography (GC) coupled with mass spectrometry (MS) and compared with reference standards enables the unambiguous identity of the major components, overall composition and can serve as a chemical fingerprint to assure the overall quality of raw materials used in various finished products. Moreover, such chemical fingerprints can be helpful to differentiate the anomalies due to possible misidentification of closely related species, the role of soil conditions, (un)intentional adulteration with other species, or contamination with extraneous ingredients.

To determine if the type of lipids present in *Limnospira* biomass and the Immulina extract could serve as potential chemical standardization markers, a GC–MS method was developed using 36 different fatty acid methyl esters as reference standards (Supplementary Fig. S1 online). As reported earlier^18,19^, the methyl esters of corresponding palmitic, linoleic, and γ-linolenic acids (γ-LA) were identified as dominant fatty acids, along with palmitoleic, stearic, and oleic acids as minor constituents in the dried biomass reference material (Supplementary Fig. S2 online). Single-step transesterification with 5% hydrogen chloride in methanol was identified as a suitable derivatization method with more reproducible results than the USP derivatization method (Table 3). In our laboratory, even though the latter method produced relative percentages of fatty acids within acceptable distribution ranges^13^, high inter-assay and intra-assay coefficients of variations (16.70% and 8.32%, respectively) were observed for the measurement of total fatty acid content. The obtained discordant results were partially attributed to the number of steps and the quality of the boron trifluoride-methanol complex utilized for the USP monogram’s derivatization method. Nevertheless, the abundance (Supplementary Fig. S2 online) of these six fatty acids was in agreement with the overall molecular composition of galactolipids mono- and digalactosyl diacylglycerol (MGDG, DGDG) and sulfolipid sulfoquinovosyl diacylglycerol (SQDG) from S*pirulina platensis* published by Xue et al.^20^ (in which palmitic, linoleic and γ-LA were identified as major fatty acids) as well as the lipid profile of *Limnospira* biomass reported in the literature^19,21–23^.

**Table 3:** Comparison of the USP method (monograph # 2181) for *Limnospira* biomass with the newly developed method.

	USP (Method A)	Newly developed (Method B)	
Analytical system	GC	GC–MS with SIM	
Derivatization method (esterification)	Two-step	Single-step	
Column	0.25 mm × 30 m fused silica capillary; 0.25 µm film of phase G16 coating	0.25 mm × 60 m fused silica capillary; 0.2 µm film of HP-88 ((88% cyanopropyl)aryl-polysiloxane) coating	
Coefficients of variation (%)	Inter-assay: 16.70 Intra-assay: 8.32	Inter-assay: 3.40 Intra-assay: 2.62	

Chemical markers	% of total (range)	% of total average ± SD (range)	Content (mg/g biomass) average ± SD (range)
Palmitic acid	35–60	47.7 ± 5.0 (38–57)	21.6 ± 2.0 (15–27)
Palmitoleic acid	2–8	4.4 ± 1.5 (2.0–9.0)	2.0 ± 0.7 (0.5–3.5)
Stearic acid	1–5	0.2 ± 0.1 (0.0–1.0)	0.1 ± 0.0 (0.0–0.5)
Oleic acid	1–7	1.5 ± 1.0 (0.0–4.0)	0.7 ± 0.5 (0.0–1.7)
Linoleic acid	13–25	20.7 ± 1.8 (14.5–24.0)	9.5 ± 1.8 (4.5–13.5)
Gamma linolenic acid	13–27	25.6 ± 5.4 (16–38)	11.9 ± 3.7 (5.5–21.0)
Alpha linolenic acid	< 0.5	Undetected	Undetected
Total fatty acids			45.7 ± 6.5 (31.0–60.0)
Utility	Identification	Identification and standardization	

Chemical marker values for the new method represent average (range) values from 32 samples.

The capillary column, J&W HP-88 (60 m × 0.25 mm i.d.), with a film thickness of 0.2 µm used in the current study, significantly increased retention, improved resolution, and reduced tailing FAMES with the GC analysis as compared to the column used in the USP method (details provided in Table 3). In our quantitation experiments, the selective ion monitoring (SIM) mode (except for methyl stearate) was identified to be at least 26–50 times more sensitive than the conventional full-scan MS mode with limits of detection (LOD) and limits of quantitation (LOQ) in 2–17 and 6–50 ppb levels, respectively (Supplementary Fig. S3 online, Supplementary Table S1 online). The selective ions, *m/z* 69 for methyl oleate; *m/z* 74 for palmitate, palmitoleate, and stearate methyl esters; *m/z* 79 for α-linolenate and γ-linolenate methyl esters; and *m/z* 81 for linoleate methyl ester were identified as suitable ions in SIM mode for enhanced detection sensitivity and quantification purposes. Together with retention indices, the SIM mode also provided good selectivity allowing unambiguous identification of the six FAMES that were detected in the TIC mode.

**Figure 2:**
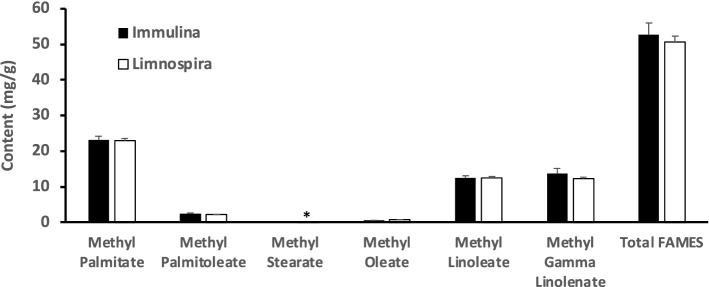
Comparative content of each FAME in dried *Limnospira* and commercial extract, Immulina, extracted from the same batch of dried biomass. Bars represent the average ± SD of three samples from the same batch of material. Each sample was analyzed in duplicate in the GC–MS. No statistically significant differences were observed between sample means, except for methyl stearate (compared by paired t-tests). *p = 0.04.

Using the developed GC–MS quantification method (Table 3), the lipid profile of the Immulina extract was investigated and compared to the dried biomass to assess whether the fatty acids in Immulina were concentrated or depleted. Only one of the minor FAME, methyl stearate, was significantly higher in the Immulina extract compared with the biomass, 0.05 mg/g versus 0.02 mg/g, respectively (*p* = 0.04, n = 3, two-tailed paired t-test). No statistically significant differences in the absolute quantities (mg/g biomass dry wt) of the other five FAMES, as well as total FAME content (mg/g biomass dry wt), was observed in Immulina compared to the corresponding biomass sourced from Dongtai Cibainian Biological Engineering, China (Fig. 2). These results indicate that the FAME profile of the biomass is preserved in Immulina and hence has significant implications for authentication of extract material. Since morphological features of botanicals are destroyed and DNA typically lost in extract material, chemical markers have emerged as a valuable tool for botanical extract authentication. The fatty acid signature can therefore not only be used as one of the methods for identification of *Limnospira* in biomass samples but also in Immulina samples (and potentially other extracts of this botanical).

To evaluate the extent of FAME variation, different batches of biomass from a single commercial grower as well as from growers throughout the world were analyzed (Supplementary Fig. S4 online).

### Assessing fatty acid profile variation in different batches of biomass from a single source

Twenty batches (acquired over the last 14 years, Supplementary Table S2 online) of dried biomass sourced from a single source were subjected to transesterification to assess the total fatty acid content as well as individual FAMES. In all these samples, the methyl ester of α-linolenic acid (α-LA) was not detected, confirming that these samples are probably free from chlorella, which usually contains α-LA at levels > 0.5% of total FAME content^24^. The total FAME content in the 20 samples ranged from 3.57 to 5.47% of biomass dry weight, with an average of 4.43%, in which polyunsaturated acids (linoleic and γ-LA) ranged from 1.32 to 3.24%. Total FAME content was significantly correlated with the date of biomass acquisition (*R*^2^ = 0.6508, *p* < 0.0001, Supplementary Fig. S5 online). Further regression analysis of the six individual fatty acids revealed that significant positive correlations were only observed for methyl linoleate and methyl γ-linolenate (*R*^2^ = 0.8036, *p* < 0.0001 and *R*^2^ = 0.5896, *p* < 0.0001, respectively, Supplementary Fig. S6 online). This data indicates that the observed decrease in FAME content with biomass age was predominantly due to the reduction of the polyunsaturated FAMES. Since polyunsaturated are more unstable than saturated fatty acids, the reduced FAME content with biomass age may be due to air oxidation of these lipids. However, controlled stability investigations are necessary before drawing major conclusions. It is possible that other factors could have contributed to the observed results. For example, the biomass was grown in outdoor ponds and as a result there could have been variation in ambient temperature during the cultivation of different batches. This may be a confounding factor since an increase in temperature^19^ has been observed to increase overall FAME content in many algal samples. Although strain differences can affect overall variations in FAME composition^18^, it is unlikely that this was a factor since all 20 samples originated from the same commercial grower.

### Assessing fatty acid profile variation in biomass sourced from 10 different countries

Accessing FAME variation in biomass obtained from different sources was achieved by analyzing product material acquired from 11 commercial growers in 10 different countries (Table 1, Supplementary Fig. S4 online). The total FAME content (sum of the 6 quantifiable fatty acids) varied between 3.14 to 5.94% of biomass dry wt (1.9-fold difference), slightly more than the variation observed for the 20 samples from a single grower (1.5-fold). However, lower content of the polyunsaturated FAMES (1.05–2.30%) was observed in the globally sourced samples. None of the samples contained detectable levels of α-LA.

### Re-evaluating the acceptance criteria for *Limnospira* fatty acid profile

The acceptance criteria range for the fatty acid profile in the USP method is broadly used for multiple species of *Arthrospira* (*A. platensis*, *A. maxima,* and *A. fusiformis*). Our sample collection included *A. platensis* and *A. maxima* (as designated by the commercial growers). Although all 32 analyzed samples are within the USP monogram’s accepted range for the percentage of palmitic acid (the predominant FAME) and linoleic acid, anomalies were observed for other FAMES (Supplementary Tables S2 and S3 online). The USP lower limit for the content of stearic and oleic acids is 1% of total FAMES. Methyl stearate was identified in negligible amounts (less than 1%) in 29 samples. Similarly, the content of methyl oleate was lower than 1% in 8 out of the 32 samples. In contrast, about 50% of samples were higher than the upper accepted criteria limit of 27% for γ-LA by 1 to 11 percentage points. Deviations from the USP accepted criteria range were also observed in our biomass reference material (0.06% of methyl stearate and 29.56% for γ-LA), highlighting the need for further revisions of this monograph.

Additionally, the acceptance criteria for the USP method only express the content of each FAME as a percentage of the total fatty acid content. A potential limitation of this approach is that two samples could have a similar profile in the percentage of each FAME but differ significantly in the absolute quantities of these compounds. For example, biomass from Chile contains the same percentage of methyl palmitate as material from Greece (49.99 and 49.21%, respectively, Supplementary Table S3 online). However, the two biomasses differed significantly in the absolute quantity of methyl palmitate (23.1 mg/g versus 15.4 mg/g, calculated from Supplementary Table S3 online). To overcome this limitation, we included total fatty acid content per gram of dry biomass as a parameter in the data analysis (values for each sample are listed in Table 1, Supplementary Tables S2 and S3 online).

### Activation of the TLR2/TLR1 signaling pathway as an in vitro bioassay for quantitation of immune-enhancing activity

Hantke and Braun^25^ identified a lipoprotein within *E. coli* that contained a unique S-glycerylcysteine residue modified with three fatty acids (palmitate and cis-vaccenate as major constituents). This lipoprotein is commonly referred to as Braun’s lipoprotein. Research over the past few decades has established the presence of these molecules in both Gram-negative and Gram-positive bacteria and determined that they are potent activators of immune cells through a Toll-like receptor (TLR)2-dependent pathway^26^. Our previous research determined that this class of lipoproteins fully accounts for the in vitro monocyte/macrophage stimulatory activity exhibited by *Limnospira*^14^. Therefore, our efforts to design a biological standardization assay for *Limnospira* focused on capturing the activity exhibited by these lipoproteins.

Selecting an appropriate in vitro bioassay for biological standardization is driven based on the product’s mechanism of action^27^. The *Limnospira*-derived lipoproteins, similar to other Braun-type lipoproteins, are activators of TLR2 signaling. This was previously demonstrated using two different approaches^28^. First, in blocking antibody studies, NF-kappa B activation by Immulina in THP-1 cells was suppressed by antibodies to CD14 and TLR2, but not by antibodies to TLR4. Second, in expression vector experiments, Immulina increased NF-kappa B-directed luciferase expression in cells co-transfected with a vector expressing proteins required for TLR2 but not TLR4 signaling. In the current research, additional supporting evidence was obtained by evaluating the activity exhibited by Immulina in cell lines engineered to stably co-express a TLR gene and an NF-kappa B inducible secreted embryonic alkaline phosphatase (SEAP) gene. Immulina exhibited a robust dose-dependent activation in cells selectively expressing TLR2, and no significant activity was detected in cells selectively expressing TLR4 at the concentrations tested (Supplementary Fig. S7 online).

**Figure 3:**
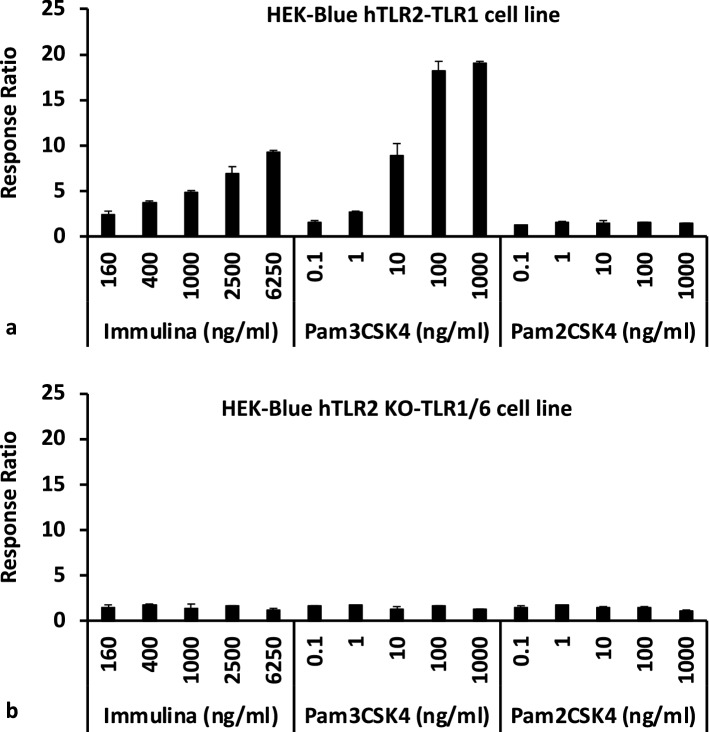
Immulina extract activates the TLR2/TLR1 signaling pathway. Activity evaluated in HEK-Blue hTLR2-TLR1 cells (**a**) and control HEK-Blue hTLR2 KO-TLR1/6 cells (**b**). Response ratio ± SD is defined as OD of sample/OD of untreated cells. Synthetic triacylated lipopeptide Pam_3_CSK_4_ was used as a positive control, and the synthetic diacylated lipopeptide Pam_2_CSK_4_ served as the negative control.

To facilitate ligand specificity and signaling, TLR2 forms heterodimers. The TLR2/TLR1 heterodimer detects triacylated lipopeptides, whereas TLR2/TLR6 heterodimer detects diacylated lipopeptides. Immulina (extracted from the biomass reference material) likely contains triacylated lipoproteins as evidenced by its ability to enhance activation in HEK-Blue hTLR2/TLR1 cells (Fig. 3a). As expected for this cell line, activation is also observed with the positive control Pam_3_CSK_4_ (a synthetic triacylated lipopeptide), but no activity was observed with the negative control Pam_2_CSK_4_ (synthetic diacylated lipopeptide). No sample induced SEAP levels above background values when evaluated in the control cells HEK-Blue hTLR2 KO-TLR1/6 cells (Fig. 3b). Cyanobacteria such as *Limnospira* have characteristics similar to gram-negative bacteria. Therefore, the presence of triacylated lipoproteins in *Limnospira* is consistent with the literature that this lipoprotein structure is also the most common type found in gram-negative bacteria^29^. Free fatty acids do not appear to contribute to the detected activity since no TLR2/TLR1-dependent activation was observed for any of the six individual fatty acids that occur within *Limnospira* (activity for individual compounds tested at 1 µg/ml ranged from 2.7 to 2.9% and were similar to untreated cells at 2.7%, values expressed as percent of activity exhibited by Pam_3_CSK_4_ tested at 100 ng/ml).

### Assessing variation in immune-enhancing activity in different batches of biomass from a single source

Based on the receptor mechanism of action for the *Limnospira* lipoproteins, the HEK-Blue hTLR2/TLR1 cell line was selected as the biological assay to quantitate immune-enhancing activity. Analysis of the 20 biomass samples obtained from a single commercial grower (Dongtai Cibainian Biological Engineering) over 14 years displayed a 27-fold variation in TLR2/TRL1-dependent activity (Supplementary Table S2 online). Similar to the chemical analysis for content of total fatty acids (Supplementary Fig. S5 online) and polyunsaturated acids (Supplementary Fig. S6 online), activity also exhibited a statistically significant decrease with sample age (*R*^2^ = 0.7644, *p* < 0.0001, Supplementary Fig. S5online). Average EC_50_ values and average total fatty acid (TFA) content for samples in the different time periods demonstrated a clear change overtime: 2021–2019 (EC_50_ 10 µg/mL, 50 µg/g TFA), 2018–2015 (EC_50_ 32 µg/mL, 44 µg/g TFA) and 2008–2007 (EC_50_ 135 µg/mL, 38 µg/g TFA). Between 2021–2019 and 2008–2007 the average EC_50_ value increased by 13.5 times, indicating that storage time was likely a major factor responsible for the 27-fold variation in activity observed in these samples.

### Assessing variation in immune-enhancing activity in biomass sourced from 10 different countries

Extracts from the 12 biomass samples sourced from commercial growers in 10 different countries exhibited a 12.5-fold variation in TLR2/TLR1-dependent activity (Table 1). Sample 6A and 6B are from the same grower and differ in activity by about threefold. This data, combined with variation observed between the 20 batches from Dongtai Cibainian Biological Engineering (Supplementary Table S2 online), indicates that there can be substantial variation between lots obtained from the same commercial supplier. Therefore, it is not valid to use the data in Table 1 to rank the companies according to producers of the highest activity biomass. Many of these commercial suppliers have proprietary strains, and a detailed analysis of many different lots would be required to evaluate whether the biomass from a particular company exhibits consistently high or low activity. Hence it remains to be determined whether the 12.5-fold difference in activity between commercial biomass samples is due to strain differences and/or environmental factors such as temperature, length of sunlight exposure, nutrient composition of growth media, etc. It is unlikely that the differences are due to biomass age since all product material was acquired during a 3-month period (November 2020 through January 2021). Evidence supporting this interpretation can be derived from the analysis of the 20 *Limnospira* samples obtained over 14 years which suggest that years (not months) are required to significantly change biomass activity (Supplementary Table S2 online).

### Content of FAMES is significantly correlated with TLR2/TLR1-dependent activity: basis for a chemico-biological approach for standardization of *Limnospira* products

The raw materials used to manufacture botanical dietary supplements exhibit inherent variability in chemical composition due to numerous factors (e.g., genetic/phenotypic variations, differences in agronomic conditions during growth, harvesting practices, etc.). This variation in raw material can influence the level of active compounds, resulting in a lack of consistent quality if the finished products are not properly standardized to the bioactives.

The *Limnospira* samples analyzed in the current study were raw materials sourced from many of the largest growers and represent biomass cultivated in diverse environments from 10 different countries. Similar to the variability observed for other botanicals^30^, our analysis of these commercial biomass samples demonstrated a twofold variation in total fatty acid content and a 12.5-fold variation in TLR2/TLR1-dependent activity (Table 1). In addition, our analysis indicated that long-term storage of biomass samples results in decreases in both activity and total fatty acid content (Supplementary Table S2 online). The public health implication of this data is that consumers are ingesting products that can vary in immune-enhancing activity by over 10 times. In addition, this level of variation poses a challenge to conducting reproducible scientific research on these products. Therefore, an effort is warranted to advance the standardization technology for *Limnospira* botanical dietary supplements and food products.

**Figure 4:**
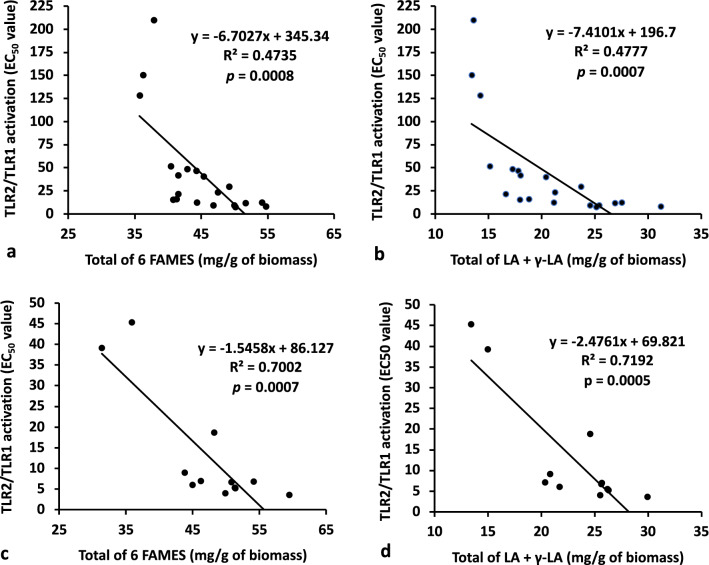
Correlations between TLR2/TLR1 extract activity and content of fatty acids in biomass material obtained from Dongtai Cibainian Biological Engineering (20 batches, **a** and **b**) and commercial growers in 10 different countries (12 batches, **c** and **d**). The total content of 6 fatty acids represents the combined total of palmitate, palmitoleate, stearate, oleate, linoleate, and γ-linolenate in each sample (**a**, **c**). “LA” and “γ-LA” represent linoleate and gamma linolenate, respectively. EC_50_ values represent the concentration (µg/mL) of biomass material required to induce activation for the TLR2/TLR1 signaling pathway to levels 50% of those achieved by Pam_3_CSK_4_ (100 ng/mL).

Regression analysis was performed to test our original hypothesis that the fatty acid profile could potentially serve as a relevant chemical standardization method to assess the relationship between total fatty acid content and immune-enhancing activity. The amount of total fatty acid (sum of the six quantifiable FAMES) was significantly correlated with TLR2/TLR1-dependent activity in the 20 biomass samples from Dongtai Cibainian Biological Engineering (*R*^2^ = 0.4735, *p* = 0.0008, Fig. 4a) as well as the 12 biomass samples from commercial growers in 10 different countries (*R*^2^ = 0.7002, *p* = 0.0007, Fig. 4c). Further regression analysis of the content of individual FAMES revealed that methyl linoleate and γ-LA were the only two compounds significantly correlated with activity in both sample sets (Table 4). The total content of the these two FAMES exhibited a similar correlation with TLR2/TLR1-dependent activity (*R*^2^ = 0.4777, *p* = 0.0007 (Fig. 4b) and *R*^2^ = 0.7192, *p* = 0.0005 (Fig. 4d), as compared to the correlation using the sum of all six FAMES. Overall, the data support our hypothesis and demonstrates that total FAME content (or just the content of methyl linoleate and γ-LA) can be used to predict the in vitro macrophage activation potential exhibited by *Limnospira* biomass samples.

**Table 4:** Linear regressions between TLR2/TLR1 extract activity and content of individual fatty acids in biomass obtained from Dongtai Cibainian Biological Engineering (20 samples, details in Supplementary Table S2 online) and commercial growers in 10 different countries (12 samples, details summarized in Table 1 and Supplementary Table S3 online).

Fatty acid	20 samples (single source)		12 samples (10 countries)	
	R-squared	*p* value	R-squared	*p* value
Methyl palmitate	0.160	0.080	**0.388**	**0.030**
Methyl palmitoleate	0.075	0.243	**0.355**	**0.041**
Methyl stearate	0.167	0.073	0.004	0.847
Methyl oleate	0.003	0.817	0.014	0.717
Methyl linoleate	**0.689**	** < 0.0001**	**0.660**	**0.001**
Methyl γ linolenate	**0.359**	**0.005**	**0.506**	**0.009**

Bold values indicate regressions that are statistically significant.

## Conclusion

In summary, we have identified an in vitro bioassay (detection of TLR2/TLR1 activity) and an analytical method (detection of FAME content) that can serve as a relevant approach for standardizing the immune-enhancing activity of *Limnospira* botanical dietary supplements and food products. Measurement of TLR2/TLR1-dependent activity detects Braun-type lipoprotein activity, the predominant compounds responsible for the in vitro macrophage activation potential of *Limnospira*. FAME content is correlated with activity and therefore expands the utility of this chemical marker to standardization efforts in addition to its original use in the USP method as a component of product identification. Utilizing the combination of an analytical method and bioassay provides an orthogonal approach to botanical standardization. The value of this combination approach has been highlighted for other botanicals such as hops (*Humulus lupulus*)^31^ and red clover (*Trifolium pratense*)^32^. Widespread adoption of chemico-biological standardization techniques to the botanical field would be expected to greatly enhance product quality and well-defined material for clinical studies on the efficacy of these products.

## Methods

### Biomass material

The biomass that was selected and taxonomically identified for reference material was obtained from Dongtai Cibainian Biological Engineering Company, LTD (established in 1994 and one of the largest producers of *Limnospira* in China). *Limnospira* dried powder (supplier lot # 20190501 and 20190820B) was used for sequence analysis. Live biomass was collected at the cultivation facility (located in Dongtai City, Jiangsu Province), and preserved in 2.5% formaldehyde for morphological analysis. The light microscopy analysis was performed on an Olympus BX53 microscope using bright field and differential interference contrast.

As part of our ongoing research on this botanical, twenty different batches of *Limnospira* dried samples were obtained from Dongtai Cibainian Biological Engineering over 14 years (2007–2021, Supplementary Table S2 online). Dried biomass samples were stored in the dark at room temperature in sealed containers at the National Center for Natural Products Research (NCNPR) and all samples were analyzed together in June and September, 2021 for fatty acid content and activation of the TLR2/TLR1 pathway, respectively.

From November 2020 through January 2021, twelve *Limnospira* dried samples were purchased from commercial growers worldwide (Table 1) that sell this biomass for food or use as a botanical dietary supplement. Biomass samples were stored in the dark at room temperature in sealed containers at the NCNPR, and all samples were analyzed together in March and May, 2021 for activation of the TLR2/TLR1 pathway and fatty acid content, respectively. Immulina extract was provided by Scandinavian Clinical Nutrition (Lot 2290006) and ChromaDex (Lots 2290020 and 2290021).

The twelve samples of powdered biomass listed in Table 1 were resuspended in distilled water and the reference sample were examined by a light microscopy for their morphological features, which were compared with known taxa listed in^16^. Subsequently their DNA was extracted using the UltraClean Microbial DNA Isolation Kit (MO BIO Laboratories, Carlsbad, CA, USA). The partial 16S rRNA gene together with the partial 16S-23S ITS region was amplified using the primers 16S27F/fD1^33,34^ and 23S30R^33^. The PCR reaction was prepared as described in^35^. The presence of the PCR products was verified on 1.5% agarose gel and subsequently sequenced or stained with Sybr Green (Lonza, Rockland, ME, USA), separated on 1.5% low melting agarose gel, excised, and cloned with pGEM-T Easy vector system (Promega Corp., Madison, WI, USA). Plasmids were purified and commercially sequenced in both directions with the use of primers T7 (5′-TAA TAC GAC TCA CTA TAG GG-3′) and SP6 (5′-TAT TTA GGT GAC ACT ATA G-3′). In case the obtained reads were not long enough to assemble into a consensus sequence of the amplified region, the internal primer CYA781F(a)^36^ was used as well. Only for samples 2, 6a, and 10 sequence reads of sufficient length and quality were obtained by direct sequencing of PCR product purified by ExoSap (Exonuclease I and FastAP Thermosensitive Alkaline Phosphatase; Thermo Fisher Scientific, Lithuania) with the use of the amplification primers 16S27F/fD1^33,34^ and 23S30R^33^. For the rest of the samples cloning needed to be used, and thus 1 to 3 clones were obtained per sample. Sequences were then submitted to the NCBI GenBank database with the accession numbers listed in Table 1. The percent identities of the 16S rRNA gene sequences were calculated in Geneious Prime 2020.2.4 (http://www.geneious.com).

### Chemistry

#### Derivatization of *Limnospira* biomass and Immulina

To characterize the overall lipid composition, dried biomass of *Limnospira* or its patented extract, Immulina, was subjected to saponification followed by esterification to convert free fatty acids into their corresponding methyl esters, hereafter referred to as fatty acid methyl esters (FAMES). All derivatization experiments (method A or method B, Table 3) were repeated at least twice, and each sample was injected twice into the GC–MS, resulting in at least quadruplicate results for every sample.

#### Saponification and esterification of biomass and Immulina (method A)

Following the USP method^13^, dried biomass from *Limnospira* and Immulina was subjected to two-step derivatization to corresponding FAMES. In brief, around 100 mg of sample was transferred to a 50 mL round-bottom flask, fitted with a reflux condenser and magnetic stir bar. To this flask, 100 mg of pyrogallic acid, 10 mL of 0.5 N methanolic sodium hydroxide solution were added and heated to reflux. After 15 min, 5.0 mL of 14% boron trifluoride—methanol was added through the reflux condenser into the round-bottom flask and continued to reflux for 2 more minutes. Around 5.0 mL of n-heptane was added through the condenser and refluxed for an additional 1.0 min. The flask was cooled to room temperature, removed the condenser, added 15 mL of saturated NaCl, closed with a stopper, shook vigorously, and the layers were allowed to separate. The supernatant *n*-heptane layer was transferred to a 2.0 mL vial, dried over anhydrous sodium sulfate, and subjected to GC–MS analysis.

#### Transesterification of biomass and Immulina (method B)

A transesterification method was developed based on earlier reports on total fatty acids in algae^37,38^. In a 20 mL scintillation vial containing 50.0 mg of biomass or Immulina, 1.5 mL of hydrogen chloride (5%) solution in methanol was added, stirred with a magnetic bar, and heated at 60 °C. After one hour, the resulting solution was cooled to room temperature, and 4.0 mL of *n*-heptane was added, stirred for 10 min, and allowed to settle as two separate layers. After 5 min, the supernatant *n*-heptane layer was carefully passed through anhydrous sodium sulfate. The resulting *n*-heptane (2.0 mL) was filtered through a 0.45 μm PVDF filter and collected in a clear glass vial for GC–MS analysis.

#### GC–MS analysis and quantitation of FAMES

##### Sample preparation

First, the samples prepared from derivatization experiments (Method A or B) were vortexed for 30 s to ensure sample homogeneity. Next, 480 µL of the sample was removed and transferred to a labeled GC vial. To the vial, 20 µL of the previously prepared internal standard (IS, methyl myristate) solution was added to the sample vial for a final concentration of 80 µg/mL. The vial was vortexed for 30 s and then analyzed by GC–MS.

##### GC/MS instrument conditions

All samples were analyzed using an Agilent 7890B gas chromatography (GC) instrument equipped with an Agilent 7693 autosampler and injector. A capillary column, J&W HP-88 (60 m × 0.25 mm i.d.) with a film thickness of 0.2 µm was connected to the GC and Agilent 5977A single quadrupole mass spectrometer (MS). Ultra-pure helium was used as the carrier gas at a flow rate of 1.2 mL/min. The samples were analyzed using the following oven program: starting from 60 °C held for 1 min, then programmed at 10 °C/min to 145 °C, then 1 °C/min to 190 °C, and finally at 5 °C/min to 240 °C. The inlet was operated in split mode at a temperature of 260 °C. The split ratio was 10:1 with 2 µL of each sample being injected. The transfer line from the GC to the MS was held at 260 °C, while the source and quadrupole were maintained at 230 °C and 150 °C, respectively, throughout the experiment.

##### Selective ion monitoring and confirmation

The 5977A mass spectrometer was operated with an electron energy of 70 eV, and the MS was operated in both scan and selective ion monitoring (SIM) modes. All scan mass spectra data were recorded at a rate of 5 Hz from *m/z* 40–400 after a 5-min solvent delay. The selective ions, *m/z* 69 for methyl oleate; *m/z* 74 for palmitate, palmitoleate, and stearate methyl esters; *m/z* 79 for α-linolenate and γ-linolenate methyl esters; and *m/z* 81 for linoleate methyl ester were identified as suitable ions in SIM mode with enhanced detection sensitivity and quantification purposes. Data were acquired utilizing Agilent MassHunter software (version B7.06.274), while quantification was achieved with Agilent MassHunter Quantitative Analysis (version 10.0.707.0). The NIST database (version 2.3) was utilized for tentative compound identification. The 6 quantifiable fatty acids were later confirmed with analytical reference standards.

##### Quantitative analyses

Methyl myristate in heptane was utilized as an IS, and the 36 reference standards (Supplementary Fig. S1 online) were obtained either from Sigma-Aldrich (St. Louis, MO USA), Thermo Fisher Scientific (Waltham, MA USA), or Cayman Chemicals (Ann Arbor, MI USA). The six quantifiable fatty acid methyl esters (FAMES) in *Limnospira* biomass, methyl palmitate, methyl palmitoleate, methyl stearate, methyl oleate, methyl linoleate, and methyl γ-linolenate were quantified using the GC–MS method. Methyl α-linolenate was also evaluated since this fatty acid was included in the USP method^13,19^ and it also served as a marker for detection of chlorella contamination^24,39,40^. A series of standard solutions were prepared for each FAME analyte to establish the calibration curves (Supplementary Fig. S3 online) using 0.1, 0.45, 0.75, 1, 4.5, 10, 25, 45, 75, 100, 200, 300, and 450 µg/mL solutions from a corresponding 5000 µL/mL solution in heptane and spiked with methyl myristate as IS with a final concentration of 80 µg/mL. Next, duplicate injections of each calibration curve solution were analyzed by GC–MS.

### Biology

#### Sample preparation for evaluation of TLR2/TLR1-dependent activity

Crude extracts were prepared from the *Limnospira* dried biomass samples to enrich the levels of Braun-type lipoproteins. Raw material (1 g) was extracted with 7 mL of 50% ethanol at 80 °C for 45 min. Following centrifugation, the supernatant was collected, ethanol concentration adjusted to 72.5% by adding 1 volume of cold 95%, and the sample incubated at − 20 °C overnight. Precipitable material was collected by centrifugation and subsequently washed with cold 95% ethanol and the final extract dried. For TLR2/TLR1-dependent activity analysis, extracts were dissolved in Milli-Q water containing 2% sodium dodecyl sulfate at 25 mg/mL.

#### Receptor cell lines

Selective detection of pathogen recognition receptors (PRR) activation was accomplished using HEK-Blue hTLR2, HEK-Blue hTLR4, HEK-Blue hTLR2-TLR1, and HEK-Blue hTLR2 KO-TLR1/6 cells (InvivoGen, San Diego CA USA). These cell lines are engineered to stably co-express a PRR gene and an NF-kappa B inducible secreted embryonic alkaline phosphatase (SEAP) gene. Activation is detected by evaluating SEAP levels in the culture medium using the QUANTI-Blue reagent with optical density measurement at 635 nm. Experiments were performed according to manufacturer instructions. Positive controls were purchased from InvivoGen and included Pam_3_CSK_4_ (TLR2/TLR1 agonist) and Pam_2_CSK_4_ (TLR2/TLR6 agonist).

## Supplementary Information

Supplementary Information.

## Data Availability

Sequencing data is deposited in the NCBI GenBank database and available online using the accession numbers listed in Table 1. All other data and materials are available upon request by contacting either Dr. Nirmal Pugh (email: ndpugh@olemiss.edu, phone: + 1 662 915 1141) or Dr. Amar Chittiboyina (email: amar@olemiss.edu, phone + 1 662 915 1572).
