# Supplementary material for: Utility of fatty acid profile and in vitro immune cell activation for chemical and biological standardization of Arthrospira/Limnospira
Source: Sci Rep. 2022 Sep 19;12:15657. doi: 10.1038/s41598-022-19590-x (PMC9485217; doi:10.1038/s41598-022-19590-x)
Supplement: Supplementary file 1 — Supplementary Information. [file 41598_2022_19590_MOESM1_ESM.pdf]

Supplementary Information

Title of the Manuscript: **Utility of fatty acid profile and *in vitro* immune cell activation for chemical and biological standardization of *Arthrospira/Limnospira***

Author List: **Jungmoo Huh, Jin Zhang, Radka Hauerová, Joseph Lee, Saqlain Haider, Mei Wang , Tomáš Hauer, Ikhlas A. Khan, Amar G. Chittiboyina, Nirmal D. Pugh**

List of Supplementary Figures and Tables:

Figure S1. GC-MS chromatogram of 36 different fatty acid methyl esters used for establishing the individual fatty acids present in dried biomass of *Limnospira* raw materials and Immulina.

Figure S2. TIC scan of FAME standards and a representative *Limnospira* sample.

Figure S3. The calibration curves along with correlation coefficient (r^2) constants for methyl palmitate (**a**), methyl palmitoleate (**b**), methyl stearate (**c**), methyl oleate (**d**), methyl linoleate (**e**), methyl γ-linolenate (**f**). and methyl α-linolenate (**g**).

Figure S4. Maximum and minimum levels of *Limnospira* FAMES in 20 batches (top) acquired from a single grower (Dongtai) and 12 lots (bottom) sourced from 10 countries.

Figure S5. Total content of FAMES (**a**) and activity (**b**) decreases with the age of biomass material.

Figure S6. Linear regressions between content of indivdual FAMES with the age of biomass material.

Figure S7. Immulina extract activates TLR2 but not TLR4-dependent signaling.

Table S1. Limits of detection (LOD) and quantitation (LOQ) levels for seven FAMES based on MS in scan and select ion monitoring (SIM) modes.

Table S2. Percentage of each FAME in 20 *Limnospira* samples as per the USP monogram acceptance criteria.

Table S3. Percentage of each FAME in 12 *Limnospira* samples from growers worldwide as per the USP monogram acceptance criteria.

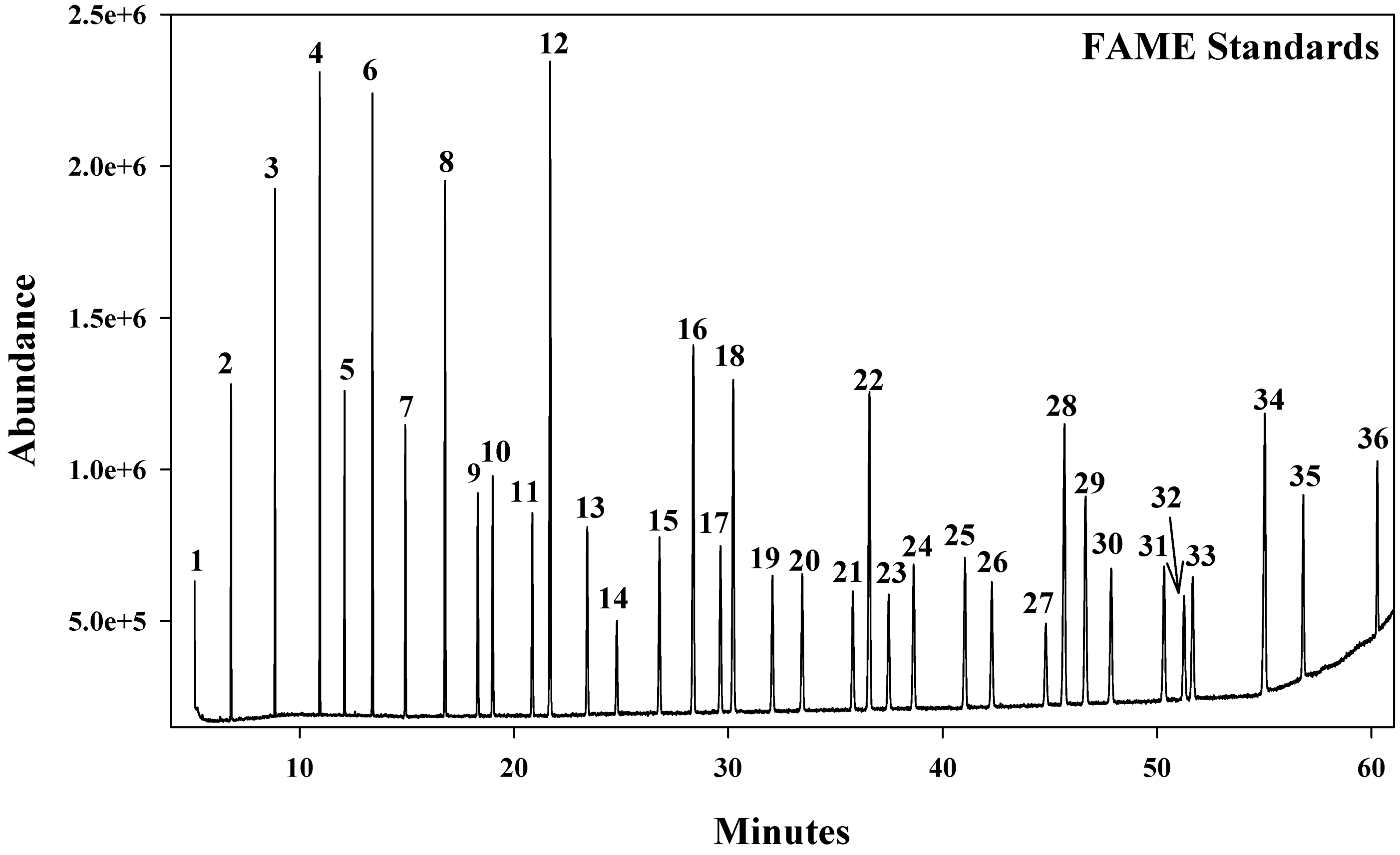

Figure S1. GC-MS chromatogram of 36 different fatty acid methyl esters used for establishing the individual fatty acids present in dried biomass of *Limnospira* raw materials and Immulina. Methyl esters of butyric (C4:0, **1**), caproic (C6:0, **2**), caprylic (C8:0, **3**), capric (C10:0, **4**), undecanoic (C11:0, **5**), lauric (C12:0, **6**), tridecanoic (C13:0, **7**), myristic (C14:0, **8**), myristoleic (C14:1, **9**), pentadecanoic (C15:0, **10**), *cis*-10-pentadecanoic (C15:1, **11**), palmitic (C16:0, **12**), palmitoleic (C16:1, **13**), heptadecanoic (C17:0, **14**), *cis*-10-heptadecenoic (C17:1, **15**), stearic (C18:0, **16**), elaidic (C18:1 n9t, **17**), oleic (C18:1 n9c, **18**), linolelaidic (C18:2 n6t, **19**), linoleic (C18:2 n6c, **20**), γ-linolenic (C18:3 n6, **21**), arachidic (C20:0, **22**), linolenic (C18:3 n3, **23**), *cis*-11-eicosenic (C20:1, **24**), heneicosanic (C21:0, **25**), *cis*-11,14-eicosadienoic (C20:2, **26**), *cis*-8,11,14-eicosatrienoic (C20:3, **27**), behenic (C22:0, **28**), inseparable [*cis*-11,14,17-eicosatrienoic (C20:3, n3) and *cis*-5,8,11,14-eicosatetraenoic (C20:4 n6), **29**], erucic (C22:1 n9, **30**), tricosanic (C23:0, **31**), *cis*-5,8,11,14,17-eicosapentaenoic (C20:5 n3, **32**), *cis*-13,16-docosadienoic (C22:2 n6, **33**), lignoceric (C24:0, **34**), nervonic (C24:1 n9, **35**)*cis*-4,7,11,13,16,19-docosahexaenoic (C22:6 n3, **36**) were used as FAME reference standards.

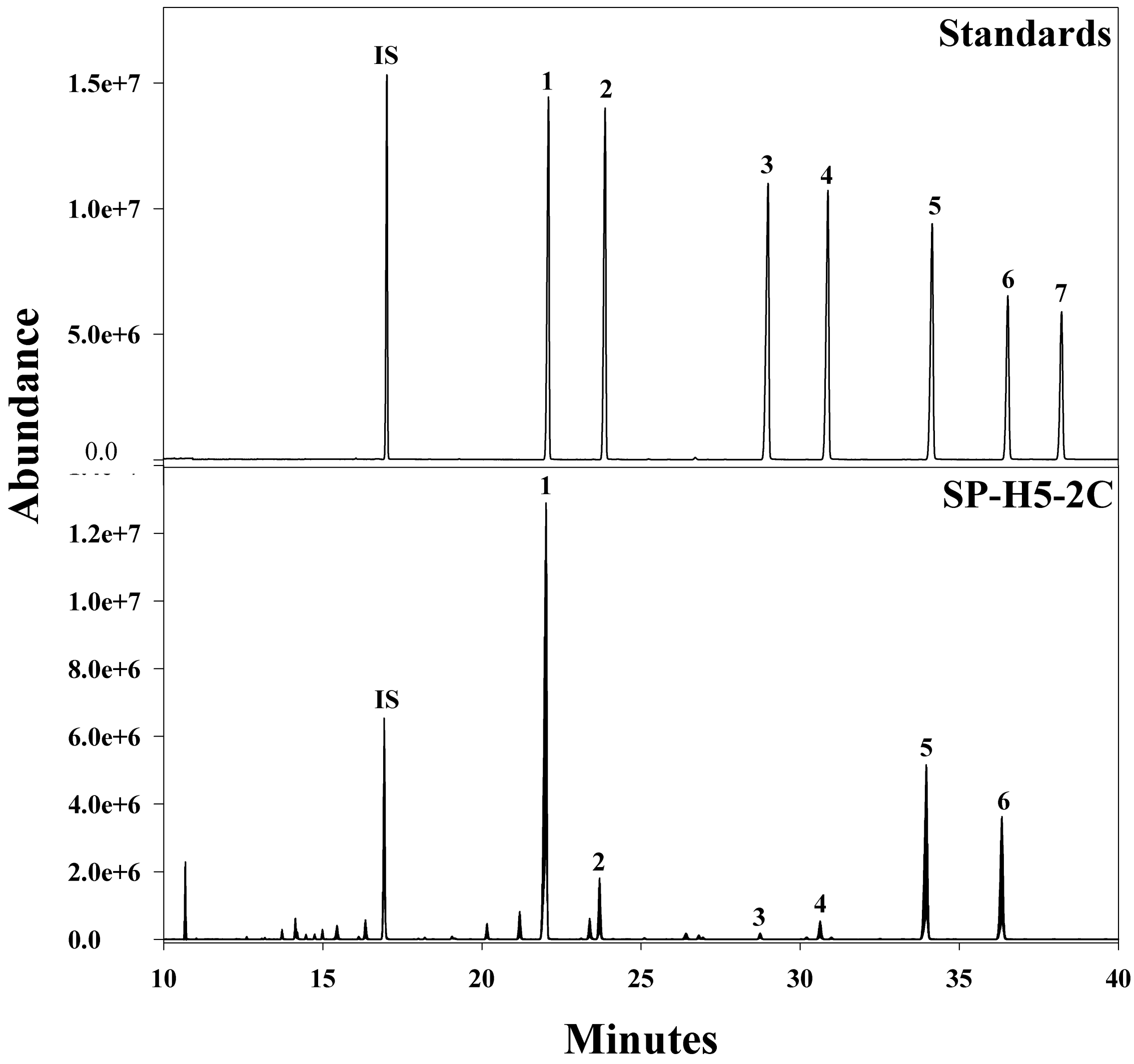

Figure S2. TIC scan of FAME standards and a representative *Limnospira* sample: **IS.** Methyl Myristate, **1.** Methyl Palmitate, **2.** Methyl Palmitoleate, **3.** Methyl Stearate, **4.** Methyl Oleate, **5.** Methyl Linoleate, **6.** Methyl γ-Linolenate, **7.** Methyl α-Linolenate.

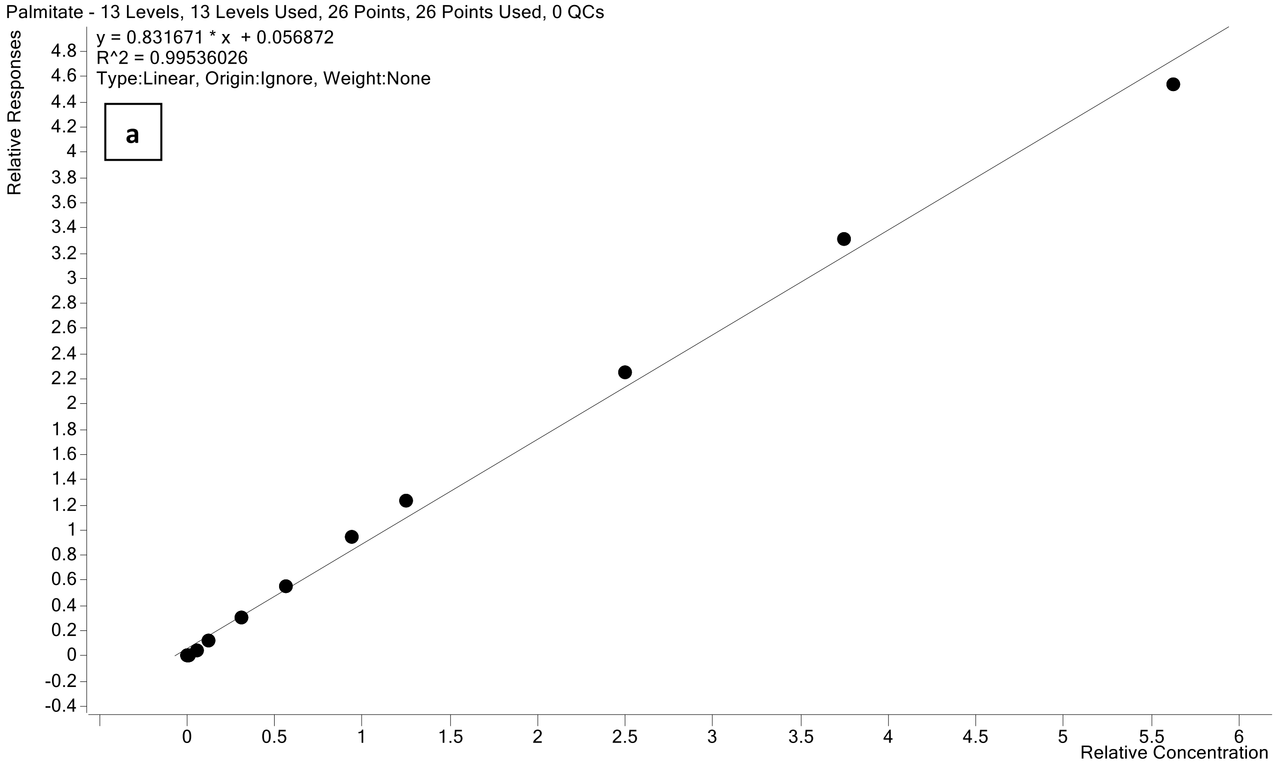

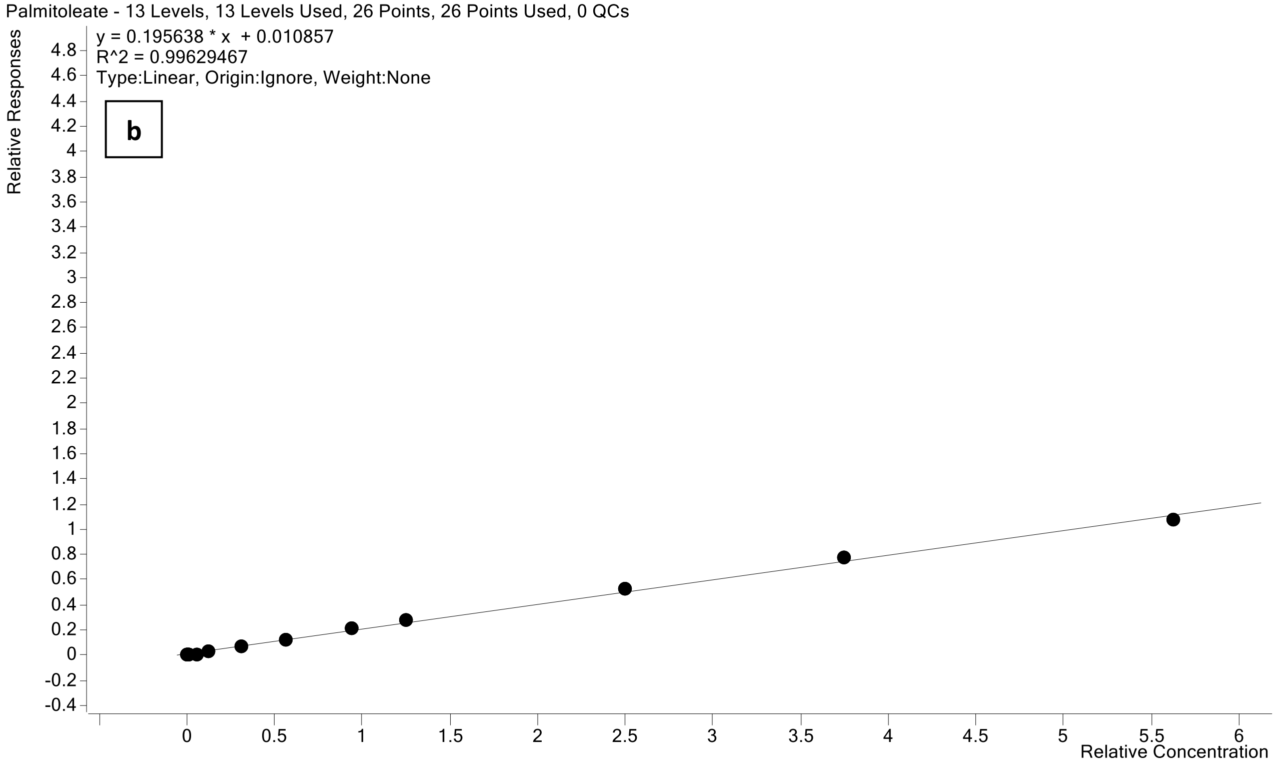

Figure S3. The calibration curves along with correlation coefficient (r^2) constants for methyl palmitate (**a**) and methyl palmitoleate (**b**).

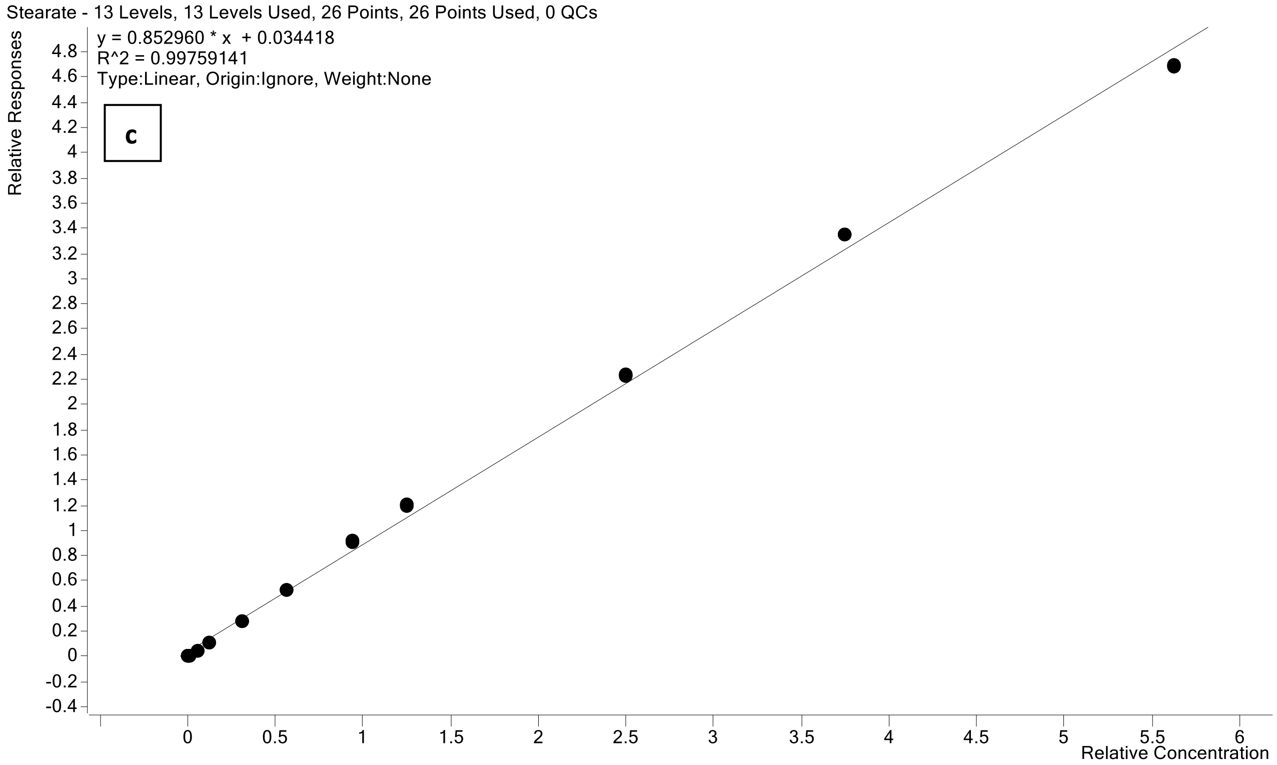

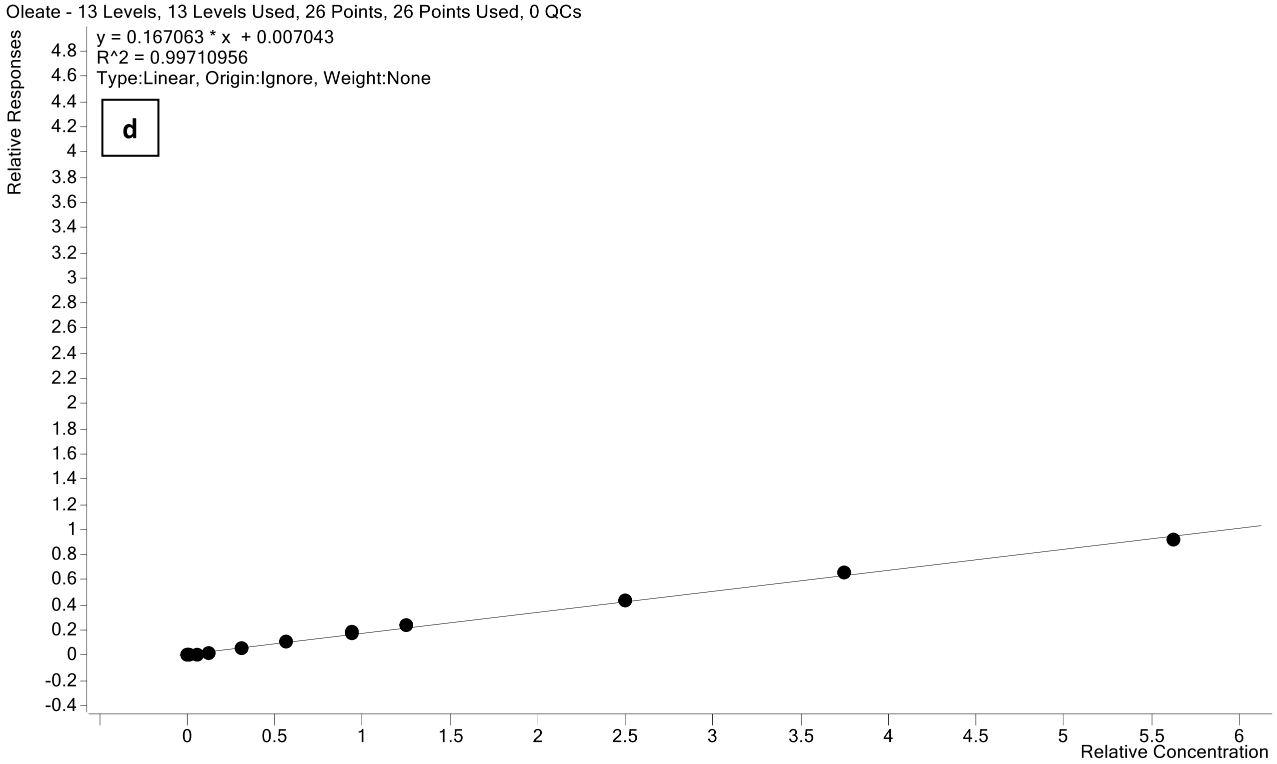

Figure S3 continued. The calibration curves along with correlation coefficient (r^2) constants for methyl stearate (**c**) and methyl oleate (**d**).

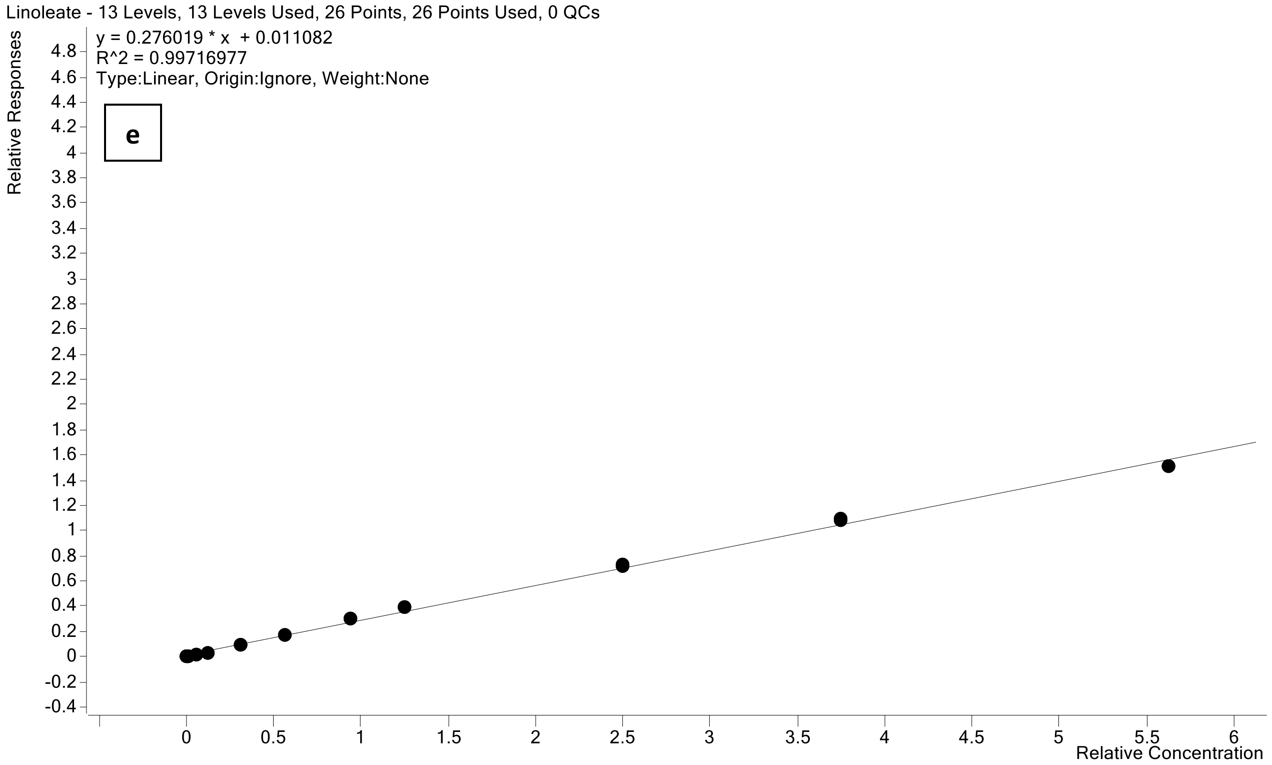

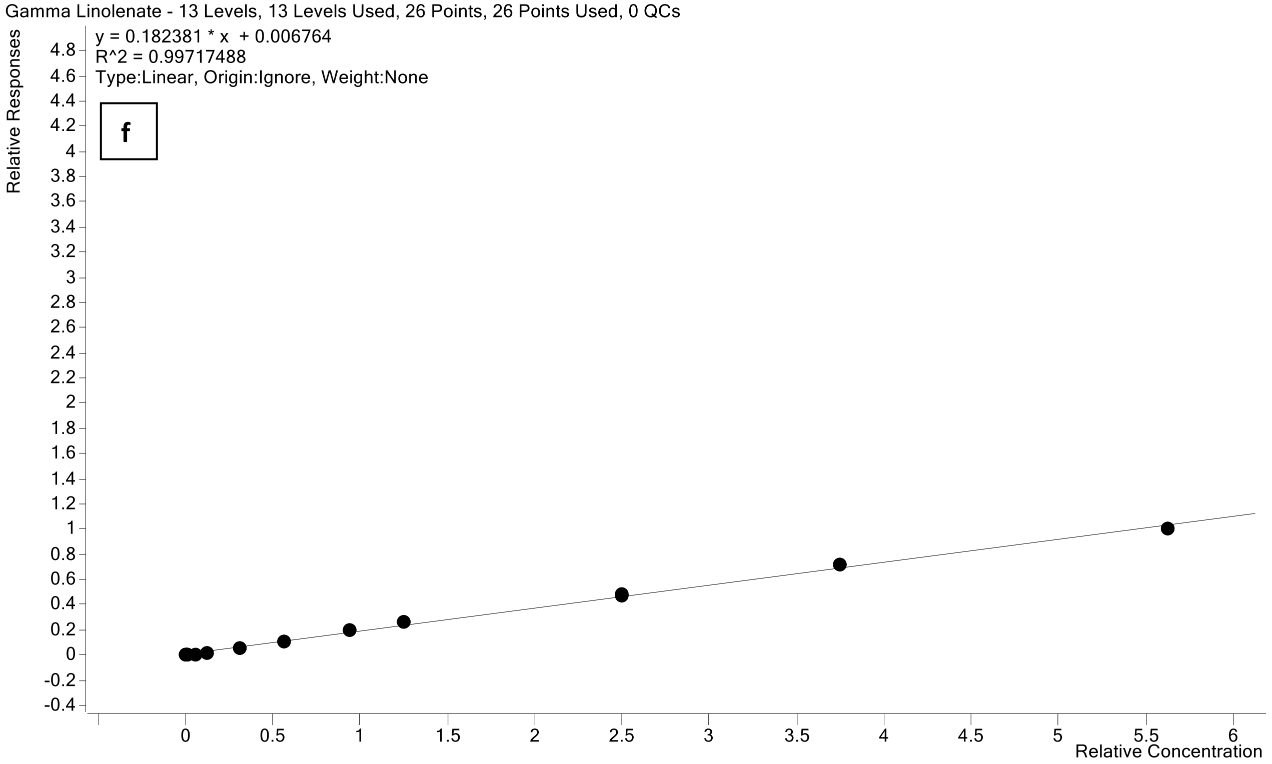

Figure S3 continued. The calibration curves along with correlation coefficient (r^2) constants for methyl linoleate (**e**) and methyl γ-linolenate (**f**).

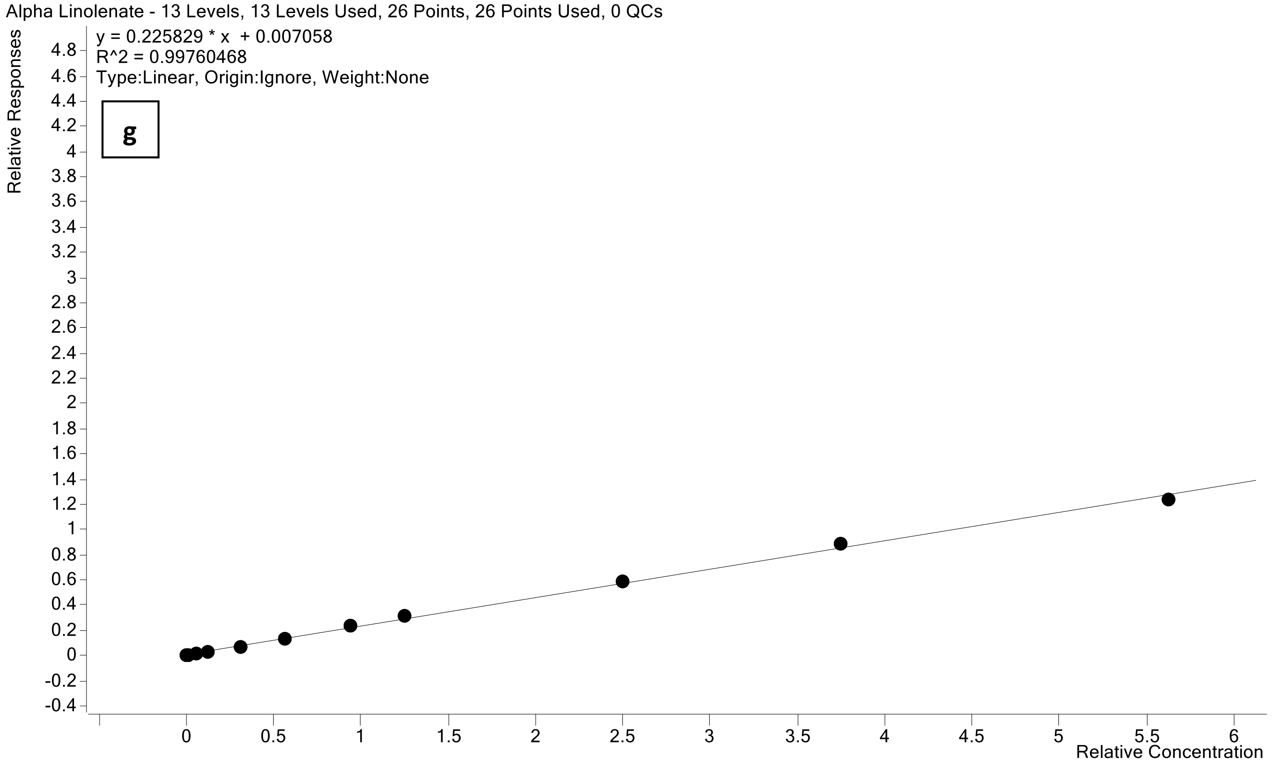

Figure S3 continued. The calibration curves along with correlation coefficient (r^2) constants for methyl α-linolenate (**g**).

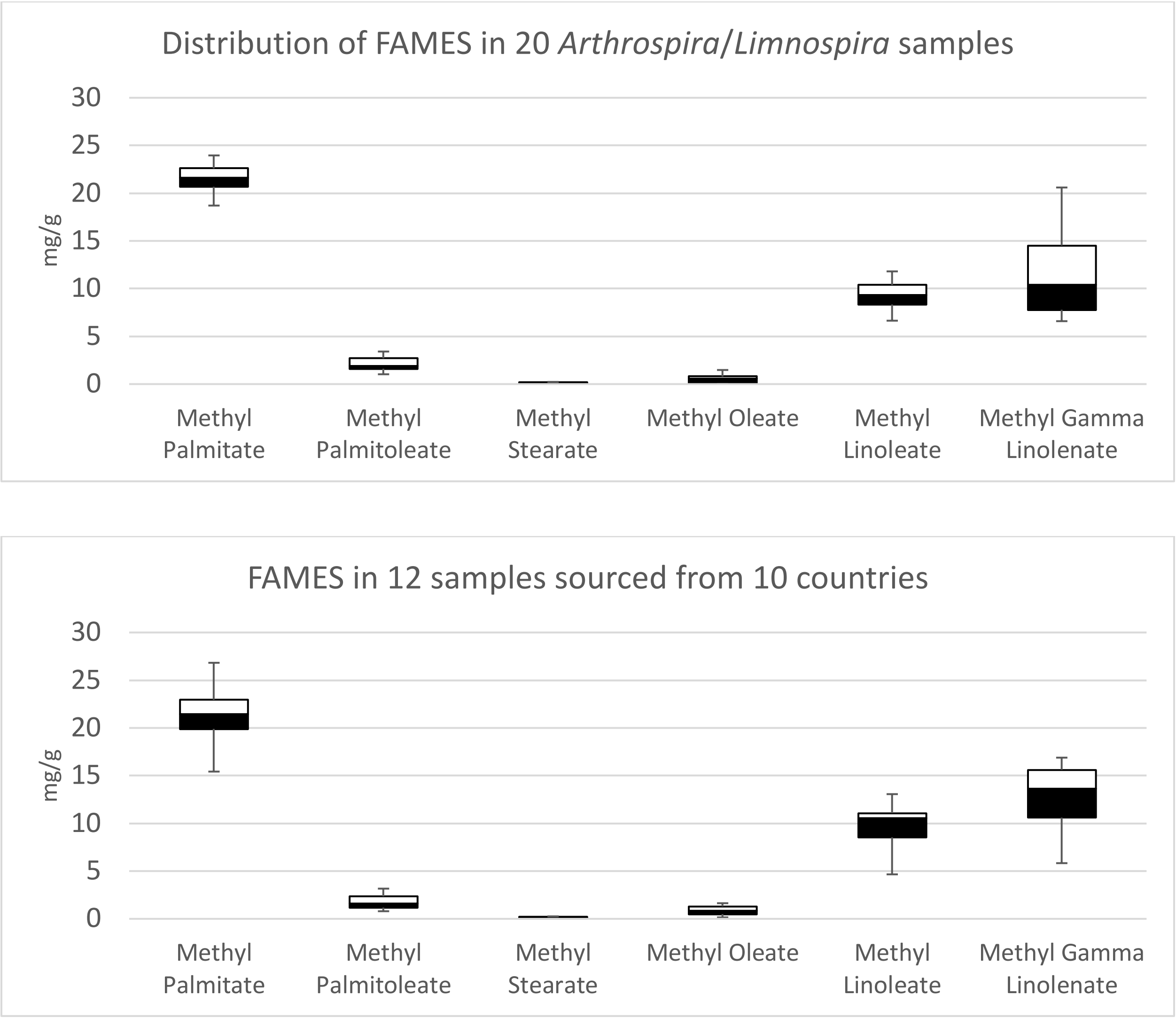

Figure S4. Maximum and minimum levels of *Limnospira* FAMES in 20 batches (top) acquired from a single grower (Dongtai) and 12 lots (bottom) sourced from 10 countries. Note: the levels of α-LA in all these samples is below LOD.

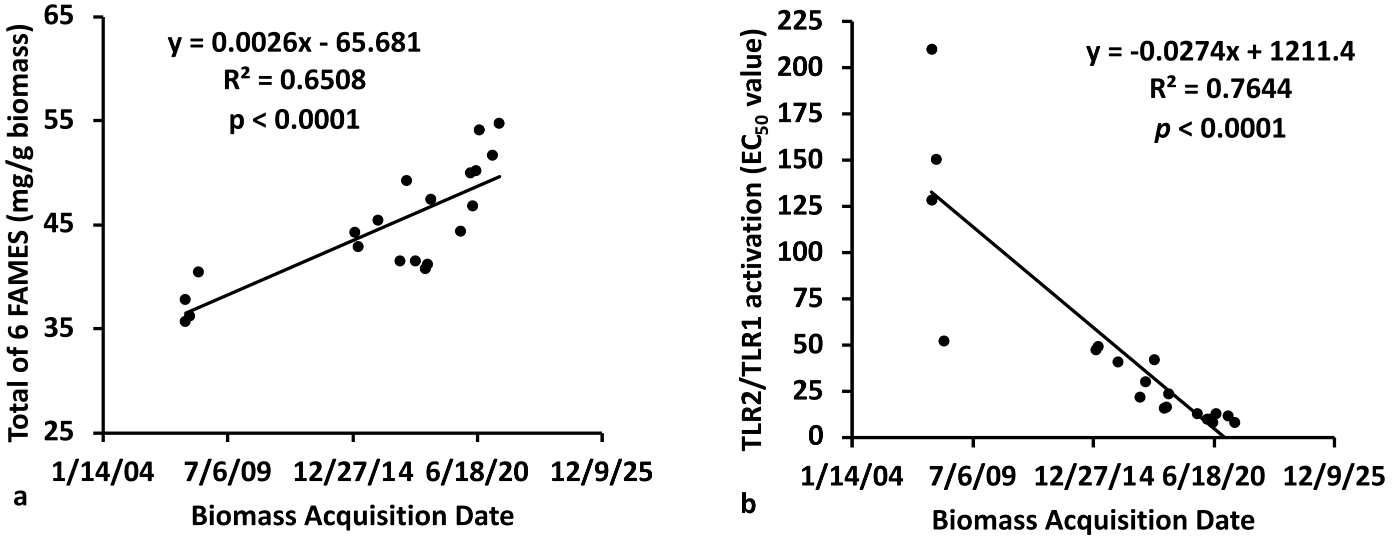

Figure S5. Total content of FAMES (**a**) and activity (**b**) decreases with the age of biomass material. The 20 samples were obtained from Dongtai Cibainian Biological Engineering, China over 14 years (dates on the x-axis refer to month/day/year). Each data point for FAME content is an average value from quadruplicate results (two independent derivatization experiments X duplicate injections for each sample). EC_{50} values for activity represent the concentration (µg/mL) of biomass material required to induce activation for the TLR2/TLR1 signaling pathway to levels 50% of those achieved by Pam_3CSK_4 (100ng/mL).

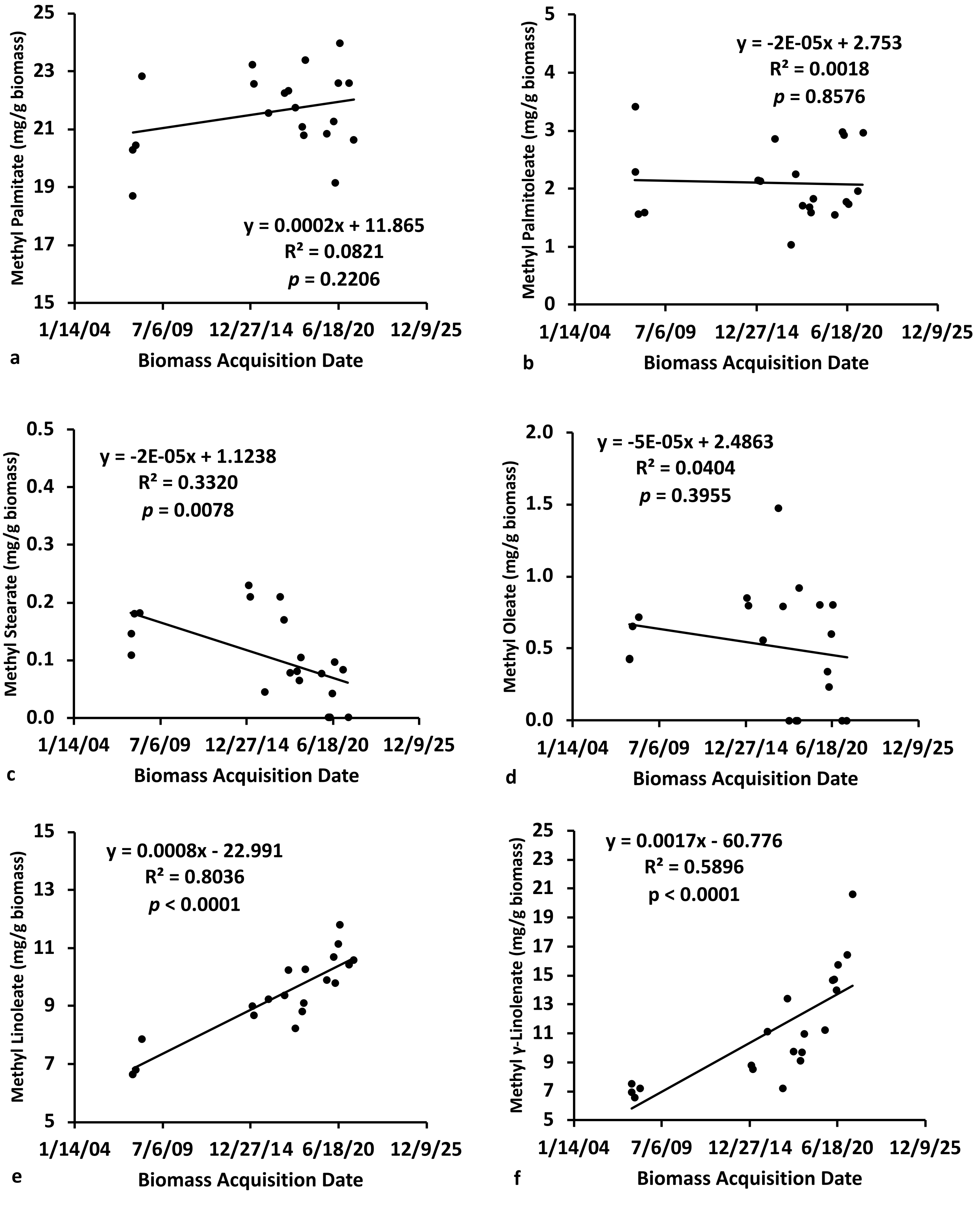

Figure S6. Linear regressions between content of indivdual FAMES with the age of biomass material. The 20 samples were obtained from Dongtai Cibainian Biological Engineering, China over 14 years (dates on the x-axis refer to month/day/year). Each data point for FAME content is an average value from quadruplicate results (two independent derivatization experiments X duplicate injections for each sample).

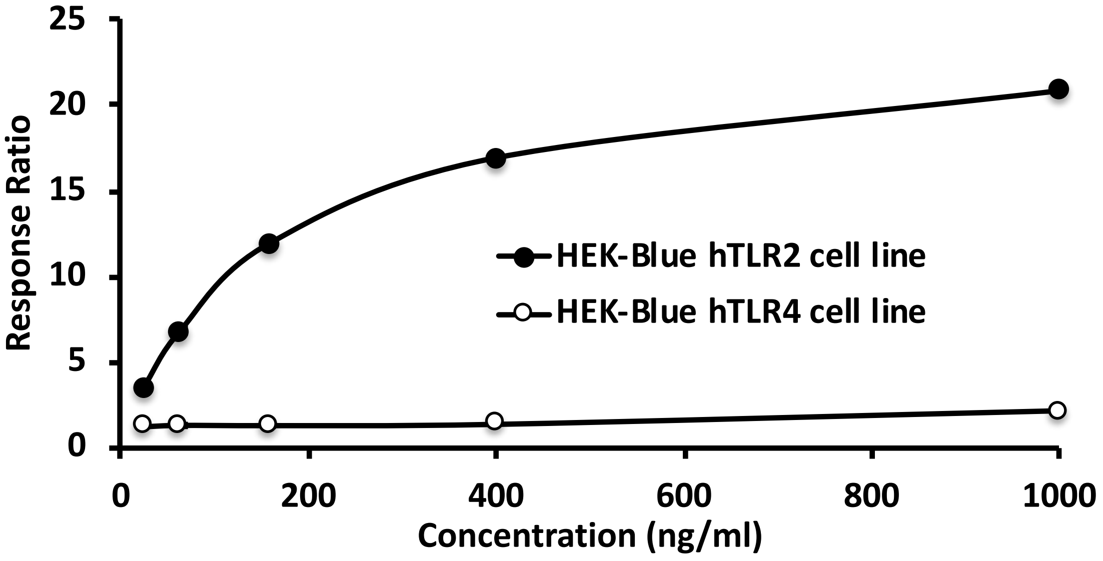

Figure S7. Immulina extract activates TLR2 but not TLR4-dependent signaling. Dose-response activity of Immulina (Lot 2290006) was determined using HEK-Blue hTLR2 and HEK-Blue hTLR4 cells. Response ratio ± s.d. is defined as OD of sample / OD of untreated cells.

Table S1. Limits of detection (LOD) and quantitation (LOQ) levels for seven FAMES based on MS in scan and select ion monitoring (SIM) modes.

FAMES	Based on Scan (ppb)		Based on SIM (ppb)		Sensitivity (SIM over MS)	
	LOD	LOQ	LOD	LOQ	LOD	LOQ
**Methyl Palmitate**	100	300	2	6	50.0	50.0
**Methly Palmitoleate**	450	1350	17	50	26.5	27.0
**Methyl Stearate**	100	300	6	17	16.7	17.6
**Methyl Oleate**	750	2250	17	33	44.1	68.2
**Methyl Linoleate**	450	1350	17	33	26.5	40.9
**Methyl γ–Linolenate**	750	2250	17	50	44.1	45.0
**Methyl α–Linolenate**	750	2250	17	50	44.1	45.0

Table S2. Percentage of each FAME in 20 *Limnospira* samples as per the USP monogram acceptance criteria, shown in parenthesis. These samples are acquired from the same source, Dongtai, China, and the levels of α-LA in these samples are below LOD and hence omitted from percentage calculations. The values outside acceptance criteria are shown in red color. EC_{50} values represent the concentration (µg/mL) of biomass material required to induce activation for the TLR2/TLR1 signaling pathway to levels 50% of those achieved by Pam_3CSK_4 (100ng/mL).

	**Samples**			**Fatty Acids Percentage of Total (%)**					
**Code**	**Date acquired**	Activity EC50 value	Total FA content (mg/g)	Methyl Palmitate (35-60)	Methyl Palmitoleate (2-8)	Methyl Stearate (1-5)	Methyl Oleate (1-7)	Methyl Linoleate (13-25)	Methyl γ-Linolenate (13-27)
**SP-JM-1**	2007_08_22	128.2	35.71	52.4	6.4	0.3	1.2	18.6	21.1
**SP-JM-2**	2007_08_23	209.9	37.86	53.6	9.0	0.4	1.1	17.6	18.3
**SP-JM-3**	2007_11_05	150.6	36.22	56.4	4.3	0.5	1.8	18.8	18.2
**SP-JM-4**	2008_03_14	52.0	40.41	56.5	3.9	0.4	1.8	19.5	17.9
**SP-JM-5**	2015_02_06	47.1	44.26	52.5	4.9	0.5	1.9	20.3	19.8
**SP-JM-6**	2015_03_!6	49.0	42.94	52.6	5.0	0.5	1.9	20.2	19.9
**SP-JM-7**	2016_02_11	40.5	45.39	47.5	6.3	0.1	1.2	20.4	24.5
**SP-JM-8**	2017_02_01	21.7	41.54	53.5	2.5	0.5	3.6	22.6	17.4
**SP-JM-9**	2017_05_09	29.9	49.22	45.4	4.6	0.3	1.6	20.8	27.3
**SP-JM-10**	2017_10_03	41.7	41.53	52.3	4.1	0.2	0.0	19.8	23.6
**SP-JM-11**	2018_03_08	15.9	40.76	51.7	4.1	0.2	0.0	21.6	22.4
**SP-JM-12**	2018_04_17	16.4	41.25	50.4	3.8	0.2	0.0	22.1	23.5
**SP-JM-13**	2018_05_29	23.3	47.48	49.2	3.8	0.2	1.9	21.7	23.1
**SP-JM-14**	2019_09_17	12.8	44.37	47.0	3.5	0.2	1.8	22.3	25.3
**SP-JM-15**	2020_03_04	9.4	49.98	42.6	6.0	0.0	0.7	21.4	29.4
**SP-JM-16**	2020_04_09	9.7	46.83	40.9	6.2	0.0	0.5	20.9	31.5
**SP-JM-17**	2020_06_01	7.9	50.16	45.1	3.5	0.1	1.2	22.2	27.9
**SP-JM-18**	2020_07_14	12.6	54.13	44.3	3.2	0.2	1.5	21.8	29.1
**SP-JM-19**	2021_02_08	11.7	51.63	43.8	3.8	0.2	0.0	20.2	31.9
**SP-JM-20**	2021_06_02	8.2	54.77	37.7	5.4	0.0	0.0	19.3	37.6

Table S3. Percentage of each FAME in 12 *Limnospira* samples from growers worldwide as per the USP monogram acceptance criteria, shown in parenthesis. These samples were acquired between November 2020 and January 2021 from commercial growers located in 10 different countries. The levels of α-LA in these samples are below LOD and hence omitted from percentage calculations. The values outside acceptance criteria are shown in red color.

Samples			Fatty Acids Percentage of Total (%)					
**Code**	Country of Origin	Total FA content (mg/g)	Methyl Palmitate (35-60)	Methyl Palmitoleate (2-8)	Methyl Stearate (1-5)	Methyl Oleate (1-7)	Methyl Linoleate (13-25)	Methyl γ-Linolenate (13-27)
**SP-CN**	USA (Hawaii)	44.91	43.74	4.02	0.31	3.62	23.89	24.41
**SP-EA**	USA (California)	59.47	45.11	2.27	0.36	1.90	21.97	28.40
**SP-SL**	Chile	46.15	49.99	3.82	0.22	1.85	21.40	22.72
**SP-TA**	Australia	35.83	55.16	2.87	0.73	3.79	21.18	16.28
**SP-FB**	Taiwan	51.30	42.20	6.17	0.06	0.59	20.09	30.90
**SP-PA**	India	50.77	44.47	2.75	0.17	2.15	21.14	29.31
**SP-TR**	India	48.15	44.82	2.33	0.11	1.73	18.27	32.73
**SP-DO**	China	51.34	41.57	6.03	0.06	1.18	21.60	29.56
**SP-NG**	Greece	31.35	49.21	2.54	0.00	0.48	14.86	32.91
**SP-FL**	Mongolia	54.10	46.64	2.49	0.46	3.02	23.56	23.83
**SP-AF**	France	43.73	46.39	4.97	0.00	1.04	19.32	28.27
**SP-AB**	Burkina Faso	49.88	41.99	4.83	0.31	1.75	22.17	28.93
